# Automatic Optimization of Lipid Models in the Martini Force Field Using *SwarmCG*

**DOI:** 10.1021/acs.jcim.3c00530

**Published:** 2023-06-06

**Authors:** Charly Empereur-mot, Kasper B. Pedersen, Riccardo Capelli, Martina Crippa, Cristina Caruso, Mattia Perrone, Paulo C. T. Souza, Siewert J. Marrink, Giovanni M. Pavan

**Affiliations:** † Department of Innovative Technologies, University of Applied Sciences and Arts of Southern Switzerland, Polo Universitario Lugano, Campus Est, Via la Santa 1, 6962 Lugano-Viganello, Switzerland; ‡ Department of Chemistry, Aarhus University, Langelandsgade 140, 8000 Aarhus C, Denmark; § Department of Biosciences, Università degli Studi di Milano, Via Celoria 26, 20133 Milano, Italy; ∥ Politecnico di Torino, Department of Applied Science and Technology, Corso Duca degli Abruzzi 24, 10129 Torino, Italy; ⊥ Molecular Microbiology and Structural Biochemistry (MMSB, UMR 5086), CNRS & University of Lyon, 7 Passage du Vercors, 69007 Lyon, France; # Molecular Dynamics, Groningen Biomolecular Sciences and Biotechnology Institute (GBB), University of Groningen, Nijenborgh 7, 9747 AG Groningen, The Netherlands

## Abstract

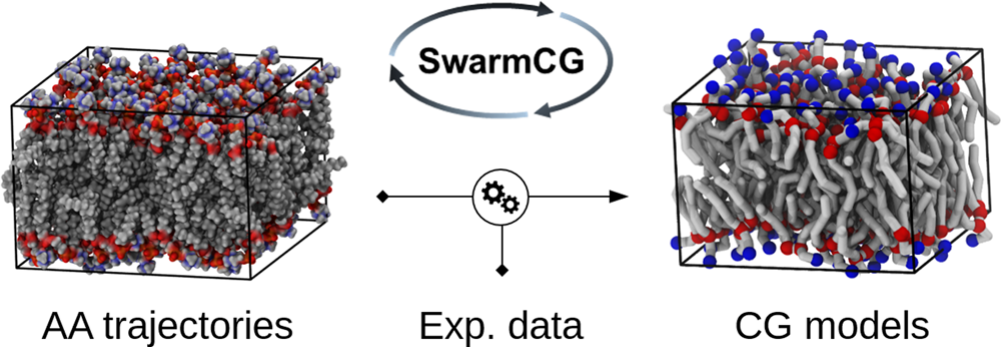

After two decades of continued development of the Martini coarse-grained force field (CG FF), further refinment of the already rather accurate Martini lipid models has become a demanding task that could benefit from integrative data-driven methods. Automatic approaches are increasingly used in the development of accurate molecular models, but they typically make use of specifically designed interaction potentials that transfer poorly to molecular systems or conditions different than those used for model calibration. As a proof of concept, here, we employ *SwarmCG*, an automatic multiobjective optimization approach facilitating the development of lipid force fields, to refine specifically the bonded interaction parameters in building blocks of lipid models within the framework of the general Martini CG FF. As targets of the optimization procedure, we employ both experimental observables (top-down references: area per lipid and bilayer thickness) and all-atom molecular dynamics simulations (bottom-up reference), which respectively inform on the supra-molecular structure of the lipid bilayer systems and on their submolecular dynamics. In our training sets, we simulate at different temperatures in the liquid and gel phases up to 11 homogeneous lamellar bilayers composed of phosphatidylcholine lipids spanning various tail lengths and degrees of (un)saturation. We explore different CG representations of the molecules and evaluate improvements *a posteriori* using additional simulation temperatures and a portion of the phase diagram of a DOPC/DPPC mixture. Successfully optimizing up to ∼80 model parameters within still limited computational budgets, we show that this protocol allows the obtainment of improved transferable Martini lipid models. In particular, the results of this study demonstrate how a fine-tuning of the representation and parameters of the models may improve their accuracy and how automatic approaches, such as *SwarmCG*, may be very useful to this end.

## 1 Introduction

Molecular dynamics (MD) has become a cornerstone tool in the study of complex molecular systems by providing high-resolution insights often inaccessible via experimental techniques. Coarse-grained (CG) molecular modeling, in which atoms are bundled together into supra-atomic particles, extends the space and time scales accessible via MD simulations and is increasingly employed to characterize systems of interest in structural biology, drug discovery, biophysics, and nanomaterials design.^1,2^ Martini^3−5^ is a popular CG modeling scheme that provides preparametrized molecular fragments (beads) for the creation of molecular models in an additive fashion. Nonbonded interactions between CG beads are described via simple spherical Lennard-Jones (LJ) and Coulomb potentials, which are parametrized according to the miscibility and partitioning of their associated molecular fragment between different solvent environments (*top-down* route), while bonded interactions are usually calibrated on the basis of equilibrium simulations of higher-resolution molecular models (*bottom-up* route). The resulting simplification of the molecular systems enables a speed up of 2 to 3 orders of magnitude with respect to equivalent all-atom (AA) modeling.^2^

The recent reparametrization of the Martini force field (FF, version 3.0.0^4^) improved the overall balance of *nonbonded* interactions between beads, as well as the accuracy of the scheme in predicting molecular packing in MD simulations. Notably, enhanced CG representation of molecular volumes can be obtained via the use of higher-resolution particles (small and tiny bead sizes). Particular attention was paid to the description of aliphatic and aromatic ringlike structures, which are ubiquitous in small molecules (e.g., solvents, drugs) and building blocks constituting macromolecules (e.g., proteins, synthetic polymers). Such improvements enable the modeling of increasingly complex systems comprising multiple classes of molecules, such as solvent mixtures, small molecules, polymers, lipid membranes, proteins, and protein–ligand systems, all within the framework of a general force field.^6−17^

In this paper, we focus on lipid models that yet remain to be updated to fully take advantage of the new interaction matrix available in Martini 3.0.0.^4^ Notably, their CG representations have remained mostly conserved since the inception of the FF^3,18^ and do not allow to differentiate between some of the lipids. This is the case, for example, of phosphatidylcholine (PC) lipids DLPC and DMPC, respectively including 12 and 14 carbons per “tail” and currently represented by identical CG models, while their phosphate-to-phosphate bilayer thicknesses (D_HH_) values differ by ∼15% at room temperature.^19,20^ The exclusive usage of big beads for modeling the fatty acids, which are designed to represent four heavy atoms and their associated hydrogens, does not provide enough resolution to differentiate between the two lipids. Further refining the CG representation of the models, in principle, will allow to further enhance thermodynamic properties of lipids and lipid mixtures in Martini simulations.^4,21^ New experimental measurements have also become available recently for lipids containing polyunsaturated fatty acids (PUFA) and should be considered for guiding the refinement of their respective models (e.g., D_HH_ thickness for SDPC and PDPC).^22^

Then, among the wide variety of thermotropic phases exhibited by saturated lipid membranes, the tilted gel (L_β′_) and ripple (P_β′_) phases are not accurately described using current Martini models,^4,23^ as bilayers preferentially adopt exclusively the straight gel phase (L_β_) at low temperatures. The further refinement of these models could focus on enabling better stabilization of the tilted gel phase (L_β′_), as well as improving the phase transition temperatures in simulations.^4,24^ Lastly, although only sparse data is available, experimental phase diagrams of lipid mixtures constitute one of the most information-rich sources usable in the calibration or validation of a FF. Maximization of the fidelity of CG simulations to phase diagrams of lipid mixtures, however, remains a challenging endeavor both in terms of FF calibration effort and computational effort (i.e., computational time, elaborate simulation setups).^4,24^ After two decades of continued Martini development, further refining the lipid models has become a demanding task that could benefit from automatized procedures and machine learning.^2^

**Figure 1:**
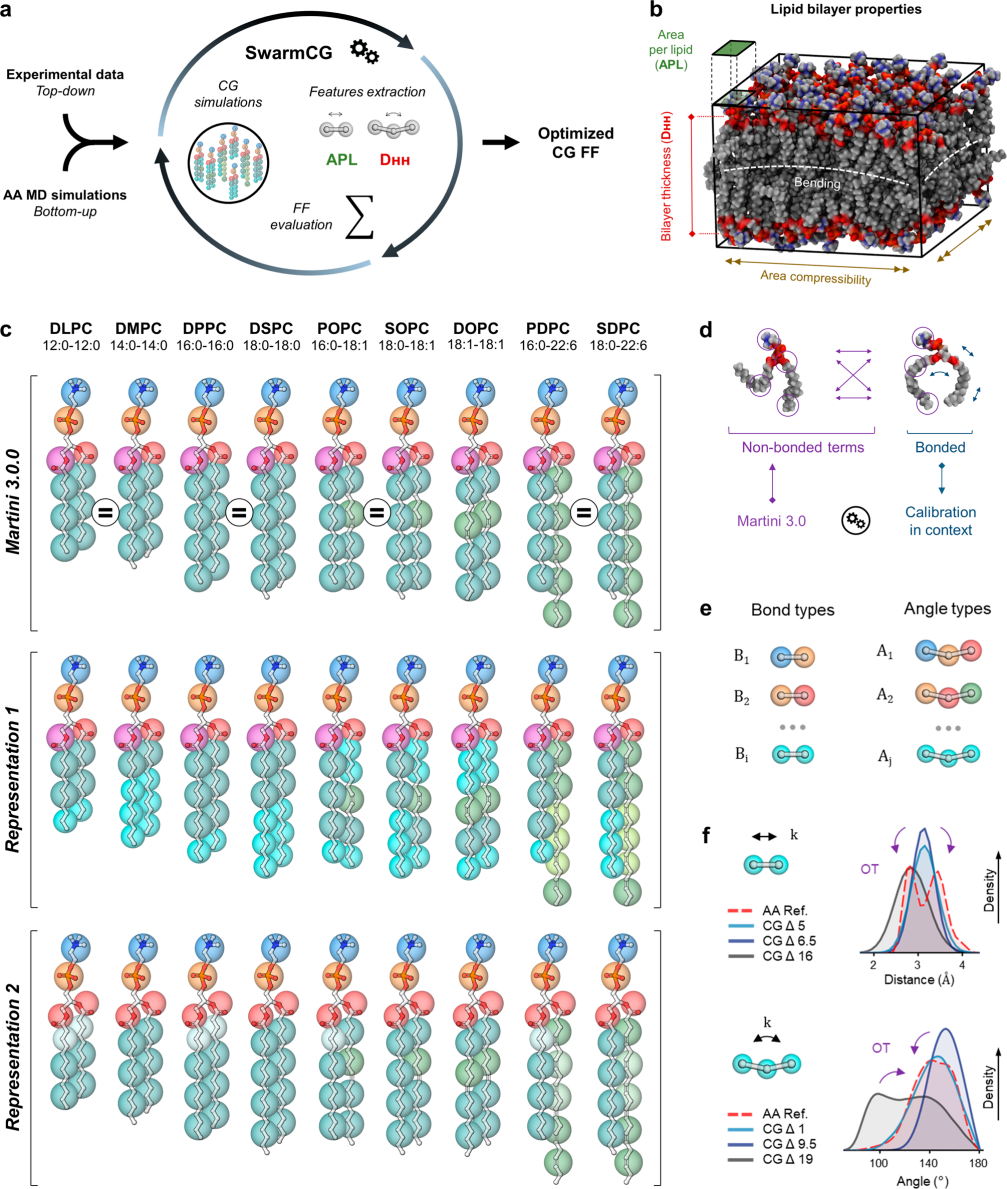
Overview of the protocol followed for obtaining *bonded* parameters via *SwarmCG* for different CG models of lipids within the framework of Martini 3.0.0. (a) *SwarmCG* simultaneously relies on *bottom-up* and *top-down* references to iteratively optimize model parameters using higher-resolution AA MD simulations and experimental data. (b) Illustration of lipid bilayer properties showing notably the APL and D_HH_ used for calculating the *top-down* component of the loss function. (c) Overview of the CG representations of interest in this study with CG beads mapping shown over AA structures using beads Q1 (dark blue), Q5 (orange), SN4a (red), N4a (purple), C1 (blue), SC2 (cyan), SC1 (white), C4h (olive), C5h (light green), and SC4h (bright yellow/green). SOPC is left out of the optimization procedures and used as part of the *posterior* evaluations. (d) Principle of the parameters calibration in this study: *bonded* parameters of the models are calibrated in the context of *nonbonded* interaction terms set to Martini 3.0.0, thereby iterating CG MD simulations of bilayers composed of different types of lipids. (e) CG bonds and angles are defined as building blocks and classified according to the CG beads they involve, which defines the type of a specific bond/angle, as well as the parameters employed. (f) Principle of the OT-B metrics used for exploiting structure-based information from AA reference simulations.

Here, we employ *SwarmCG*,^25,26^ an automatic multiobjective optimization approach that facilitates the development of transferable lipid FFs, to evaluate and compare the potential of two putative refined CG representations of the Martini lipids. Using simultaneously in the training sets eight PC lipids simulated at different temperatures and spanning different phase states, we optimize the *bonded* parameters in building blocks of the lipid models, while *nonbonded* parameters remain constant and set to Martini 3.0.0^4^ (Figure 1a–d). The application of *SwarmCG*(25,26) here guarantees that an optimal set of *bonded* parameters has been found for each resolution compared according to a given set of molecular modeling constraints (i.e., composition of the training set, *nonbonded* parameters, and a limited number of other simulation parameters). This eliminates uncertainties related to parameters tuning, which in turn allows for the gaining of insights on the relative ability of each CG representation to further enhance the thermodynamic properties of the lipid models in the Martini framework. The two putative CG representations are compared *a posteriori* using a range of temperatures, an additional PC lipid type that is not included in the training set, and by simulating a portion of the phase diagram of a DOPC/DPPC mixture known to simultaneously exhibit two phase states experimentally.^24,27^

## 2 Methodology

### 2.1 Automated Optimization of CG Lipid Models

The optimization protocol proposed in *SwarmCG*(25,26) allows for the obtainment of CG FF parameters for lipid models by simultaneously exploiting experimental data (*top-down*: area per lipid and D_HH_ thickness) and AA MD simulations (*bottom-up*: bond and angle distributions), which, respectively, inform on the supra-molecular structure of the systems and on their submolecular dynamics (Figure 1a,b, Sections S1 and S2, and Tables S1 and S2). CG models are tested iteratively in a set of simulations of small patches of homogeneous lamellar bilayers used to measure the discrepancies observed between the simulated and reference data (128 lipids and 200 ns of equilibrium CG MD simulation, each—Section S3, Table S3). The software allows for the modulation of selected parameters of the CG FF that are iteratively optimized within predefined boundaries using FST-PSO^28^ (Fuzzy Self-Tuning Particle Swarm Optimization, one of the most efficient PSO variants to date^29^) for the minimization of a loss function designed to improve FF accuracy.

The quality and completeness of the information embedded in the training set directly conditions the accuracy of the output FF, as well as its ability to generalize to different types of lipids and to different thermodynamic conditions. In this study, because the *bonded* parameters are defined independently of temperature as general building blocks that are redundant across lipid types while at the same time the space of their potential solutions is restrained by other simulation parameters remaining constant (notably *nonbonded* parameters set to Martini 3.0.0^4^), the use of a rich training set can be expected to output a consistent CG lipid FF that generalizes well to other types of lipids and to other observables than those used as objectives during optimization^26^ (Section S1). The CG representations of the lipids, however, might still limit the extent to which reference data can be fitted, depending on the degrees of freedom preserved by the molecular models (i.e., depending on the choice of beads describing *nonbonded* interactions, their reference positioning, the topology of the CG models, and the potentials used to describe *bonded* interactions).

In the following experiments, we use in the training sets eight PC lipids spanning tails of different lengths and different degrees of (un)saturation, for which experimental measurements are available for pure composition lamellar bilayers in the liquid and gel phases (area per lipid and D_HH_ bilayer thickness).^19,20,22,30^ Among the corresponding models in Martini, the following pairs are currently represented identically: DLPC and DMPC (12:0–12:0 and 14:0–14:0), DPPC and DSPC (16:0–16:0 and 18:0–18:0), POPC and SOPC (16:0–18:1 and 18:0–18:1), and SDPC and PDPC (16:0–22:6 and 18:0–22:6) (Figure 1c). This is also the case for their respective head-type variants: DLPE and DMPE, DLPS and DMPS, etc. To resolve this issue, we explore two different CG representations of the lipids that allow for the unique mapping of each molecule, the experimentation of the use of multiple bead sizes to preserve degrees of freedom in the models, and the allowance of *SwarmCG*(25,26) to precisely accommodate the reference data.

### 2.2 Loss Function

The loss function, to be minimized, reformulates our many-objective optimization problem into a single-objective one by aggregating the distances from both *top-down* and *bottom-up* objectives into a global “FF accuracy score.” Fitting of the APL and D_HH_ experimental data is formulated as the two primary objectives that have equal importance (*top-down* global features), while reproduction within the CG models of the distributions of bond and angle values calculated from AA MD simulations constitutes a secondary objective (*bottom-up* local features) that is less emphasized. To this end, we apply a soft penalty whose role is to ensure large deviations from the reference *bottom-up* data are forbidden and allow for effective distribution in between the *bonded* building blocks of the residual error inherent to the coarse-graining process (i.e., inherent to the reduction of the number of degrees of freedom in between AA and CG models). The loss function is defined as
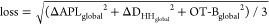
1where ΔAPL_global_ and ΔD_HH_global__ are the aggregated percentage deviations from experimental data, calculated across the training set as
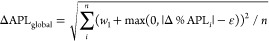
2and
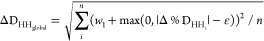
3where Δ%APL_*i*_ and Δ%D_HH_*i*__ are the percentage deviations observed on average within the *i*^th^ simulation of a given pair of lipid type and temperature included in the training set; ε represents the tolerated measurements error in Δ%APL_*i*_ and Δ%D_HH_*i*__ (set to 1.5); *w*_1_ is a weight used to prioritize fitting the *top-down* objectives over the *bottom-up* objectives (set to 10), which means the protocol is allowed to discard structure-based information for better fitting experimental measurements; and *n* is the number of pairs of lipid types and temperatures included in the training set.

For the calculation of the *bottom-up* component of our loss function (OT-B_global_ in eq 1), as preliminary steps, we obtain well-sampled AA MD trajectories for each bilayer included in the training set at the temperatures selected for optimization (128 lipids and 1 μs of equilibrium AA MD simulation, each). We employ the Charmm 3.6^31^ AA FF, which has demonstrated good accuracy for the simulation of PC lipids, in general, and particularly for saturated PC lipids in the gel phase^32^ (Section S2, Table S1). We then project (map) these trajectories according to our CG representations of interest (Figure 1c, Section S3) and compute all AA-mapped bond and angle distributions to be used as reference during optimization. The bond and angle types are defined, respectively, as all possible connected pairs and triplets of beads, which maps atomic connectivity into beads connectivity according to our CG representations (Figure 1c,e). We evaluate the mismatch between corresponding CG versus AA-mapped bond and angle distributions using the Wasserstein distance^33,34^ (a.k.a., Earth mover’s distance, EMD), a metric based on optimal transport^35^ (OT, Figure 1f), which has several useful properties: (i) multimodal distributions are well handled, (ii) distances are robust to noise, (iii) distances are quantified in interpretable units (e.g., Å, degrees), and (iv) their computations are inexpensive (via PyEMD^33,36^). This metric (hereafter referred to as “OT-B metrics”) has been already proven well-suited for parametrizing the *bonded* terms of CG models of complex and flexible molecules in a previous version of *SwarmCG*.^25^ The *bottom-up* component of the loss informs on how closely a putative set of FF parameters allows matching of the AA description of the molecular systems included in the training set and is calculated as
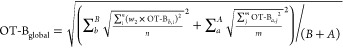
4where OT-B_*a,i*_ and OT-B_*b,j*_ are the OT-B distances calculated respectively for the *i*^th^ instance of bond type *b* and the *j*^th^ instance of angle type *a*, with *n* and *m*, respectively, as the number of instances of bond type *b* and angle type *a* in all simulations included in the training set and *B* and *A*, respectively, as the number of bond and angle types appearing in the CG models included in the training set (Figure 1e). The weight *w*_2_ (set to 50) allows for the obtainment of comparable OT-B values whether the metrics are applied on bonds or angles and prioritizes adjusting first the distributions of the bonds. When *w*_2_ is set to 50, a distance of 0.4 Å between the distributions of two bonds is considered equivalent to a distance of 20 degrees between the distributions of two angles. When *w*_1_ is set to 10, fitting of the experimental measurements is prioritized as long as OT-B distances are below 0.2 Å for bonds and below 10 degrees for angles (on average and for all bond and angle types). This setting of *w*_1_ also provides the required flexibility for adequately exploiting even slightly inaccurate AA-MD reference data (Section S2). The convergence criterion is defined as 10 swarm iterations without improving loss.

## 3 Results

### 3.1 Representation 1: Mixed-Tail Resolution Helps Improve Fidelity in the Liquid Phase

**Figure 2:**
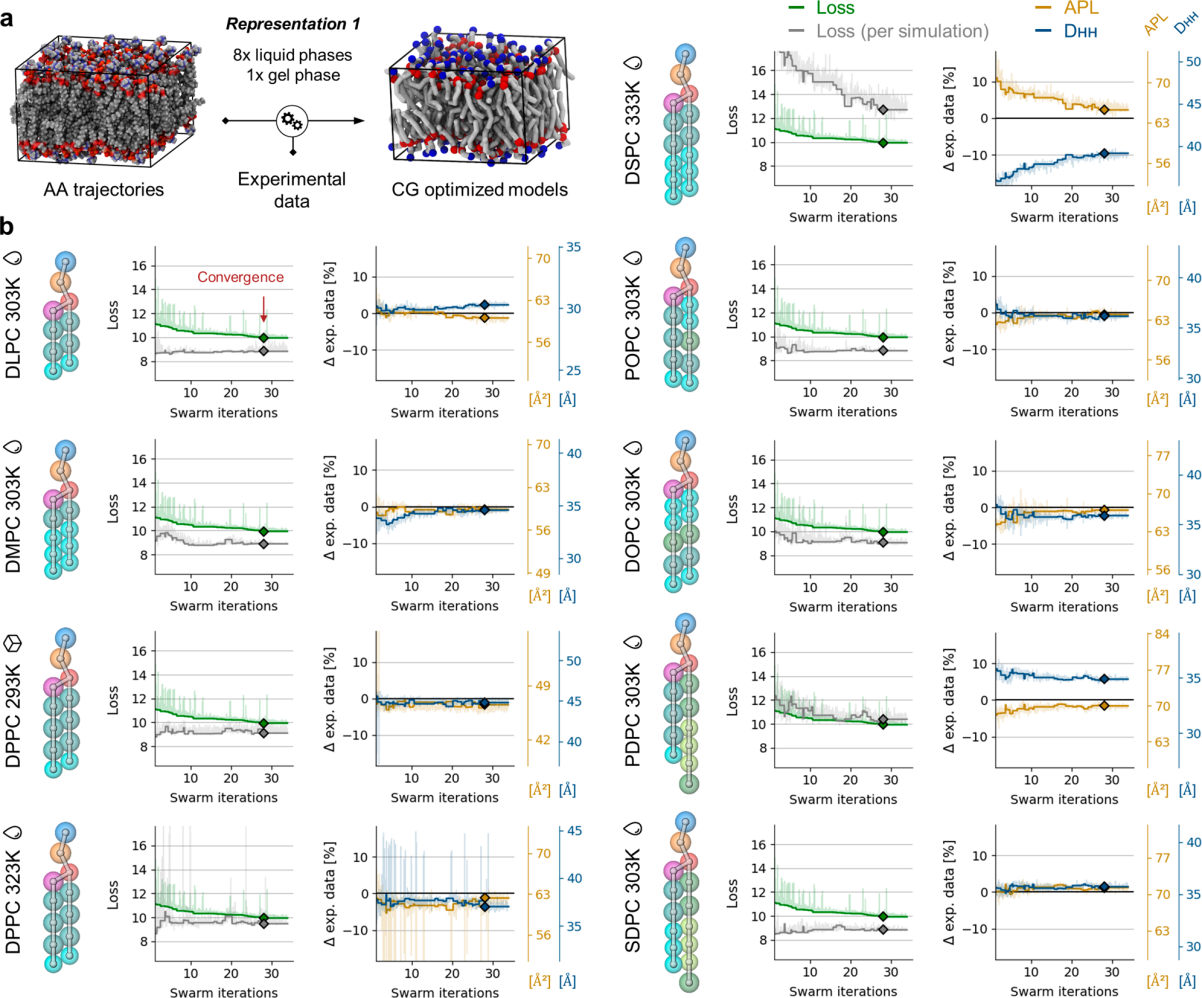
Multiobjective optimization of the *bonded* parameters of the FF for PC lipid models built in the framework of Martini 3.0.0 using Representation 1 and in the training set bilayers of 8 different lipid types simulated at nine temperatures (DLPC, 303 K; DMPC, 303 K; DPPC, 293 and 323 K; DSPC, 333 K; POPC, 303 K; DOPC, 303 K; PDPC, 303 K; and SDPC, 303 K). (a) Illustration summarizing the workflow. (b) Left panels: loss global (green) and loss per bilayer simulation (gray) in the training set. Right panels: APL (yellow) and D_HH_ (blue) for each bilayer simulation in the training set. The horizontal black lines set at 0 identify the target experimental APL and D_HH_ values. Solid curves are values corresponding to the best global loss at any point during optimization. Shaded lines show raw data. Diamonds represent values at convergence obtained with the optimized *bonded* parameters. The drop and box icons, respectively, represent the liquid and gel states of pure lipid bilayers at the corresponding temperatures.

As a first experiment, we employ *SwarmCG*(25,26) to calibrate the *bonded* parameters in building blocks of PC lipid models using Representation 1 (Figure 1c, 2a, Section S4), including in the training set lipids spanning tails of various lengths and degrees of unsaturation. The hypothesis motivating this CG representation is that mapping the lipid tails as precisely as possible by using regular beads to represent exactly four heavy atoms and small beads to represent exactly three heavy atoms may allow an enhanced description of their flexibility and dynamics (N.B., exclusively regular beads were used to represent lipid tails thus far, and Martini 3.0.0^4^ offers well-calibrated smaller beads). We build lipid tails according to the following arbitrary rules, by order of priority: (i) minimize number of beads; (ii) include only one unsaturation per bead, ideally a regular one; and (iii) stack regular beads at the start of the lipid tails. We optimize model parameters by iteratively simulating nine different patches of lamellar bilayers, thereby having each of the eight lipid types simulated in the liquid phase and only the DPPC bilayer simulated also in the tilted gel phase (L_β′_) at 293 K. In order to limit bias in the evolution of the systems toward either the liquid or gel phase, both DPPC simulations start from a configuration in the ripple phase (P_β_′, Section S3). We calibrate equilibrium values and force constants for all bonds and all angles defined in the lipid models, which totals 77 parameters across 16 bond types and 27 angle types (Figure S1, equilibrium values remain set to 180 deg for nine angle types). The swarm of particles is initialized randomly within the 77-dimensional search space, except for the first particle that is provided with knowledge from AA-mapped MD simulations (initialized using average equilibrium values computed for all bond and angle types) and from previous Martini lipid models^4^ (initialized within relevant ranges of force constants).

The steady decrease of the global loss (Figure 2b, green curve) indicates the *bonded* parameters of the models are adapted successfully and allow approaching of the objectives until the optimization converges at swarm iteration 29. At convergence, the models overall correctly reproduce the APL and D_HH_ experimental measurements defined as target (Figure 2b, yellow and blue curves converging toward reference black line, set to 0), and only the D_HH_ values for DSPC and PDPC in the liquid phase remain meaningfully improvable (N.B., here, we calculate the phosphate-to-phosphate bilayer thickness to approximate the D_HH_). This is also visible when calculating the loss separately for each simulation (Figure 2b, gray curves), which additionally indicates that fitting the objectives is harder for DSPC at 333 K than it is for PDPC at 303 K (Figure 2b, gray curves remain over green curves) in the context of the modeling choices of this experiment (CG representation, composition of the training set, etc.). The OT-B distances are otherwise minimized effectively, which indicates that the structural features present in the reference AA-mapped MD trajectories are overall well reproduced in the CG descriptions of the systems (Figures S2–S4). In terms of computational time, here, the refinement of 77 *bonded* parameters of the lipid models using nine informative simulations required 15 days (wall-clock time) to reach convergence (39 swarm iterations) using 27 particles in the swarm and using 64 CPU cores (each CG simulation running on a single CPU core and scaling horizontally by parallelizing the swarm of particles on an inexpensive CPU machine).

**Figure 3:**
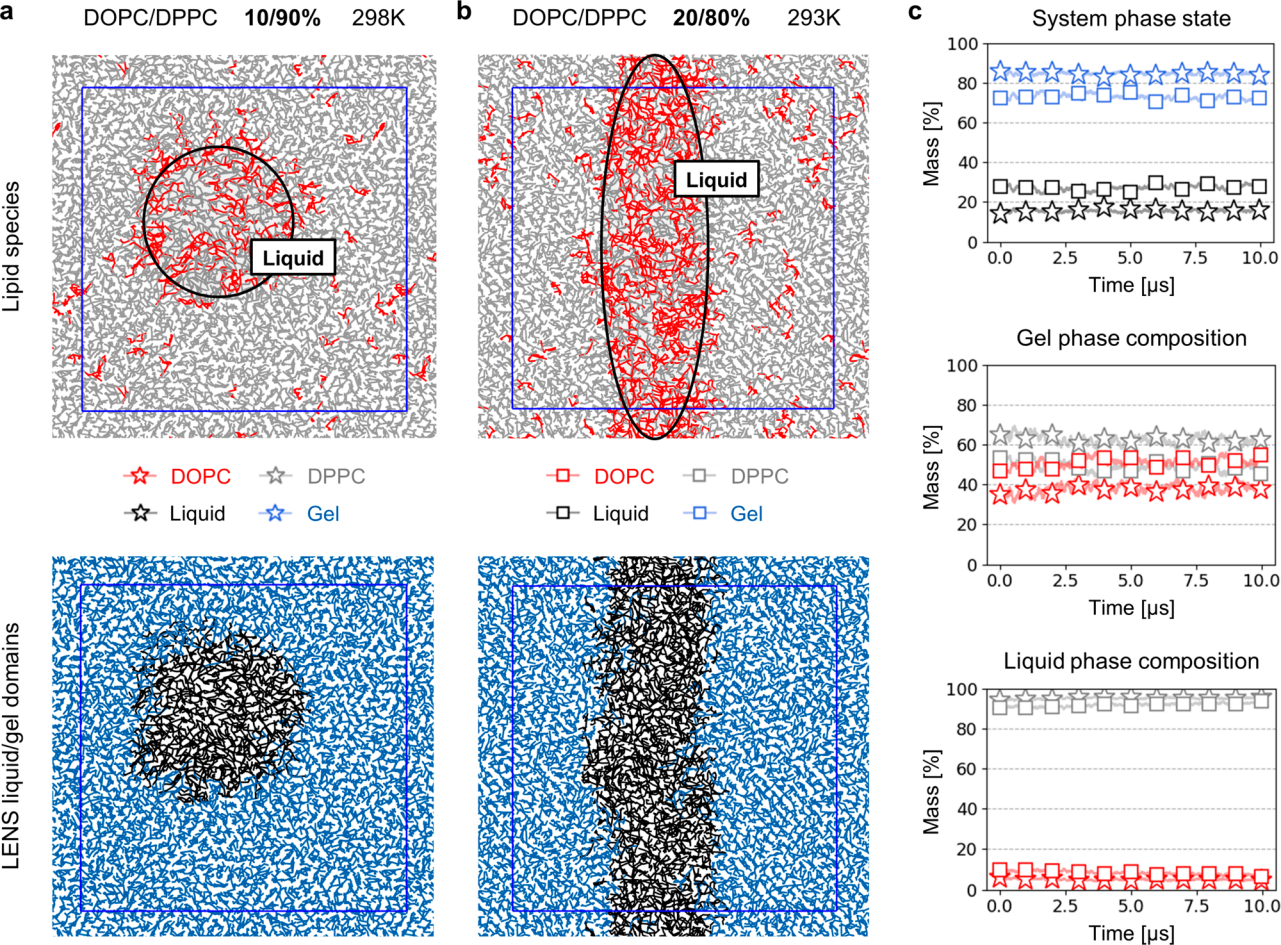
Characterization of the phase separation in DOPC/DPPC mixtures with lipid models using Representation 1. (a) Orthogonal view of a bilayer composed of 1152 lipids at 10/90% mass of DOPC/DPPC. Top: colored according to lipid type (red, DOPC; gray, DPPC). Bottom: colored according to phase state using LENS (blue, gel phase; black, liquid phase). (b) Orthogonal view of a bilayer composed of 1152 lipids at 20/80% mass of DOPC/DPPC. Top: colored according to lipid type (red, DOPC; gray, DPPC). Bottom: colored according to phase state using LENS (blue, gel phase; black, liquid phase). (c) Top: mass percentage of the system in the gel (blue) and liquid (black) phase across simulations at 10% (stars) and 20% (squares) mass DOPC. Middle: mass percentage of DOPC (red) and DPPC (gray) found in the gel phase across simulations at 10% (stars) and 20% (squares) mass DOPC. Bottom: mass percentage of DOPC (red) and DPPC (gray) found in the liquid phase across simulations at 10% (stars) and 20% (squares) mass of DOPC.

To further estimate the balance of *bonded* and *nonbonded* interactions in these models, we evaluate *a posteriori* their ability to describe phase separation in DOPC/DPPC mixtures known to simultaneously exhibit two phase states at 298 K^24,27^ (this phase separation is not correctly described using Martini lipid models version 3.0.0). Simulating a bilayer composed of 1152 randomly dispersed lipids at 10/90% mass DOPC/DPPC and starting from a configuration in the liquid phase, the nucleation of the gel phase is observed after ∼100 ns of equilibration, and the equilibrated system exhibits a liquid/gel phase separation after ∼1 μs (Figure 3a). To characterize the phase separation, we employ the LENS (Local Environments and Neighbors Shuffling) descriptor,^37^ which allows for the local evaluation of the changes in the environment of a molecule across trajectory frames and, here, enables the classification of lipids into phase states. The proportion of lipids in the gel and liquid phases is stable in an additional 10 μs of production simulation, during which the two phases constantly exchange lipids (Figure 3c, stars; Supplementary Movie 1). Reiterating this experiment with 20/80% mass DOPC/DPPC in the system, we do not observe gel phase nucleation after 1 μs of equilibration. When the simulation temperature is lowered to 293 K, stable phase separation can be observed also at 20/80% mass DOPC/DPPC after ∼1 μs of equilibration (Figure 3b), again with a constant exchange of lipids in between the two phases (Figure 3c, squares).

Altogether these results indicate that an optimal solution was found for parametrizing the *bonded* terms of Martini lipid models using the alternative Representation 1, according to the objectives defined. Structural properties appear improved for the selected lipids in the liquid phase and in the gel phase specifically for DPPC, the *bonded* parameters having been calibrated in order to obtain an optimal compromise specifically to this end. However, because Representation 1 makes use of repeated small and big beads for modeling saturated tails (Figure 1c), the lateral packing of the tails cannot be correctly described in the gel phase notably for DMPC and DSPC. In particular, when tested *a posteriori* the transition in between liquid and gel phases is not observed for DMPC and DSPC bilayers are not stable enough in the gel phase (Figure 5). Repeating this parametrization experiment and also including in the training set simulations of DMPC and DSPC bilayers in the gel phase (273 and 308 K, respectively) does not allow for further improvement of the models. Instead, this additional experiment shows that no satisfying solution can be found for parametrizing toward these objectives the *bonded* parameters of lipid models using the philosophy of Representation 1 (Figure S5).

### 3.2 Representation 2: More Homogeneous Tail Resolution Balances Improvement across Phases

**Figure 4:**
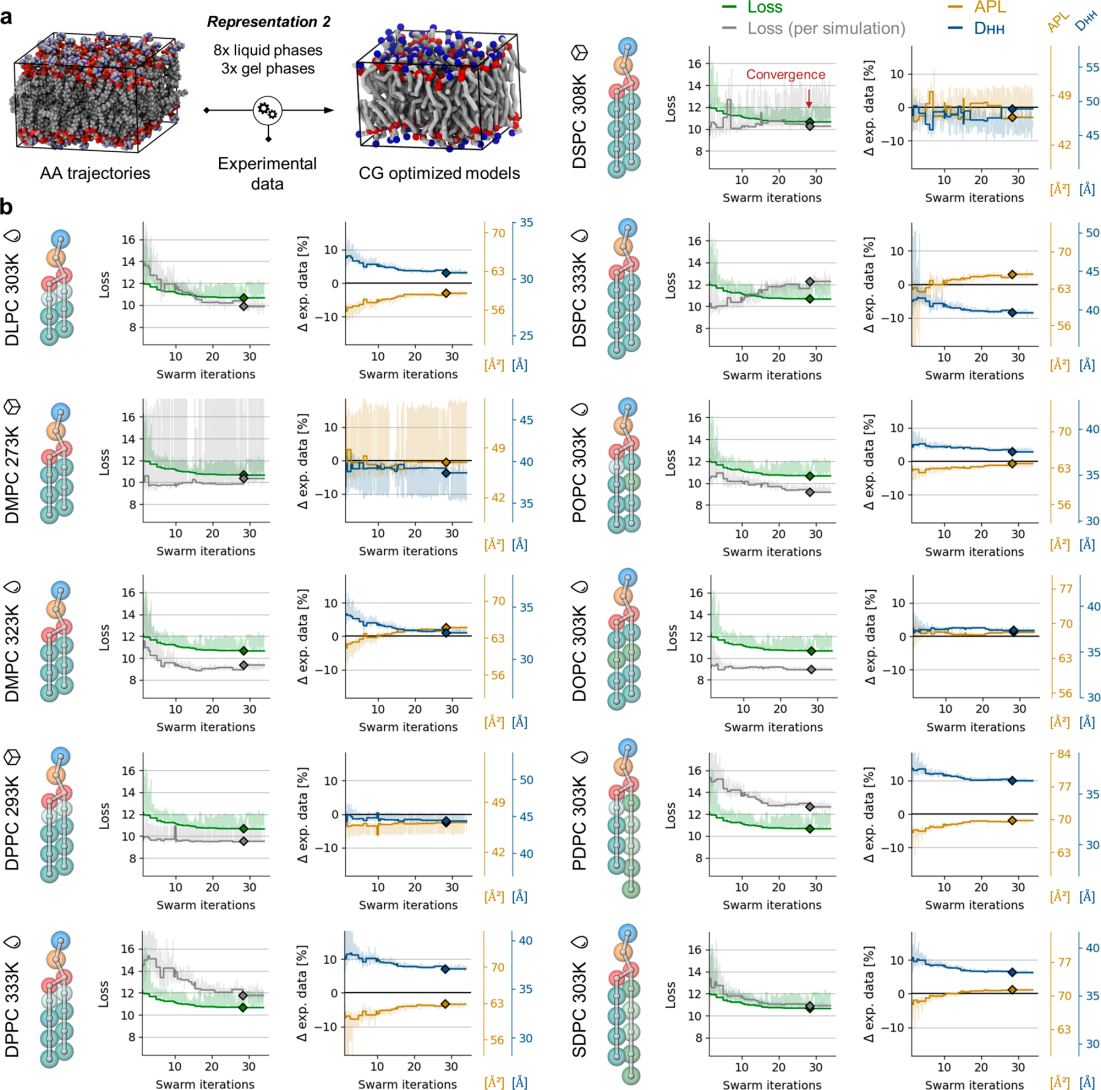
Multiobjective optimization of the *bonded* parameters of the FF for PC lipid models built in the framework of Martini 3.0.0 using Representation 2 and in the training set bilayers of 8 different lipid types simulated at 11 temperatures (DLPC, 303 K; DMPC, 273 and 303 K; DPPC, 293 and 323 K; DSPC, 308 and 333 K; POPC, 303 K; DOPC, 303 K; PDPC 303 K; and SDPC 303 K). (a) Illustration summarizing the workflow. (b) Left panels: loss global (green) and loss per bilayer simulation (gray) in the training set. Right panels: APL (yellow) and D_HH_ (blue) for each bilayer simulation in the training set. The horizontal black lines set at 0 identify the target experimental APL and D_HH_ values. Solid curves are values corresponding to the best global loss at any point during optimization. Shaded lines show raw data. Diamonds represent values at convergence obtained with the optimized *bonded* parameters. The drop and box icons, respectively, represent the liquid and gel states of pure lipid bilayers at the corresponding temperatures.

We then adapt our protocol and perform an automated search of an optimal set of *bonded* parameters for calibrating the building blocks of lipid models using Representation 2 (Figures 1c, 4a, and Section S5). The hypothesis motivating this alternative representation is that while attempting to better preserve degrees of freedom during the coarse-graining process, the use of exclusively regular beads in the bulk of all saturated tails remains mandatory for preserving the thermodynamic properties of the lipids in both the liquid and gel phases (N.B., tails are differentiated by using for next-to-ester groups either a small bead with 3-to-1 mapping or a regular bead with 5-to-1 mapping). We include in the training set the same eight PC lipids as in the previous experiment, for which we simulate patches of lamellar bilayers in the liquid phase at the same temperatures as before, but this time, we directly include bilayer simulations in the tilted gel phase (L_β′_) for DMPC, DPPC, and DSPC (273, 293, and 308 K, respectively). This provides more information for guiding the optimization and introduces additional constraints enforcing the transferability of the *bonded* building blocks across phase states. Here, also, to limit bias in the evolution of the system toward either the liquid or gel phase, DMPC, DPPC, and DSPC simulations start from a configuration in the ripple phase (P_β_′, Section S3). We calibrate equilibrium values and force constants for all bonds and all angles defined in the lipid models, which totals 48 parameters across 13 bond types and 12 angle types (Figure S6, equilibrium values remain set to 180 deg for two angle types). The swarm of particles is initialized randomly within the 48-dimensional search space, except for the first particle that is provided with knowledge from AA-mapped MD simulations (initialized using average equilibrium values computed for all bond and angle types) and from previous Martini lipid models (initialized within relevant ranges of force constants known to be adequate).

The steady decrease of the global loss (Figure 4b:, green curve) indicates the *bonded* parameters of the models are adapted successfully until the optimization converges at swarm iteration 28. The presence of noise in the evaluation of the APL and D_HH_ thickness for DMPC at 273 K and DSPC at 308 K indicates that the sets of parameters explored during optimization do not systematically trigger the formation of a gel phase (Figure 4b, light yellow and blue curves; Section S3). At convergence, the models overall correctly approach the APL and D_HH_ experimental measurements defined as target (Figure 4b, yellow and blue curves converging toward reference black line, set to 0), which indicates Representation 2 allows for description of the thermodynamic properties of various types of lipids. The optimization problem now includes more and different constraints on the *bonded* parameters that are introduced by adding in the training set the simulations of DMPC and DSPC in the tilted gel phase (L_β′_), as well as by reducing the number of beads in the lipid tails, notably for PUFA-containing lipids (i.e., slightly lower resolution than Representation 1). The APL is very well fitted to experimental data for all pairs of lipid type and simulation temperature, both in the liquid and gel phases, while the D_HH_ thicknesses remain slightly less fitted on average. The OT-B distances here also are minimized effectively, thereby reproducing overall within the CG models the structural features present in the reference AA-mapped MD trajectories (Figures S7–S9).

The error on fitting D_HH_ experimental data remains the largest for DPPC and DSPC at 333 K and for PDPC and SDPC at 303 K (Figure 4b, blue curves reaching a plateau away from the reference black line). These four lipids have saturated tails in common, including 16 and 18 carbons (PDPC and DPPC both include 16:0 tails, and SDPC and DSPC both include 18:0 tails), which indicates compromises had to be made during the optimization of the *bonded* parameters of these tails for matching experimental data set as the target in different phase states and for the highly flexible PUFA-containing lipids. In the context of this rich training set, which is representative of the variety of PC lipids and includes transversal experimental data, this result indicates there exists no set of *bonded* parameters that allow for further improvement of the matching of D_HH_ thicknesses for these lipid models using Representation 2 (in the context also of the other optimization and modeling constraints: ranges defined for the exploration of equilibrium values and force constants, bonds and angles defined in the CG representation, *nonbonded* parameters, and other simulation parameters used). Calculation of the loss separately for each simulation shows that fitting the objectives was harder particularly for DSPC at 333 K and PDPC at 303 K (Figure 4b, gray curves remain over green curves). When the previous simulation protocol of a DOPC/DPPC mixture at 10/90% mass for Representation 2 is repeated, we can observe gel/liquid phase separation after adapting the temperature to 288 K (10 K below the experimental reference). In terms of computational time, here, the refinement of 48 *bonded* parameters of the lipid models using 11 informative simulations required 13 days (wall-clock time) to reach convergence (38 swarm iterations) using 23 particles in the swarm and using 64 CPU cores (each CG simulation running on a single CPU core, scaling horizontally by parallelizing the swarm of particles on an inexpensive CPU machine).

**Figure 5:**
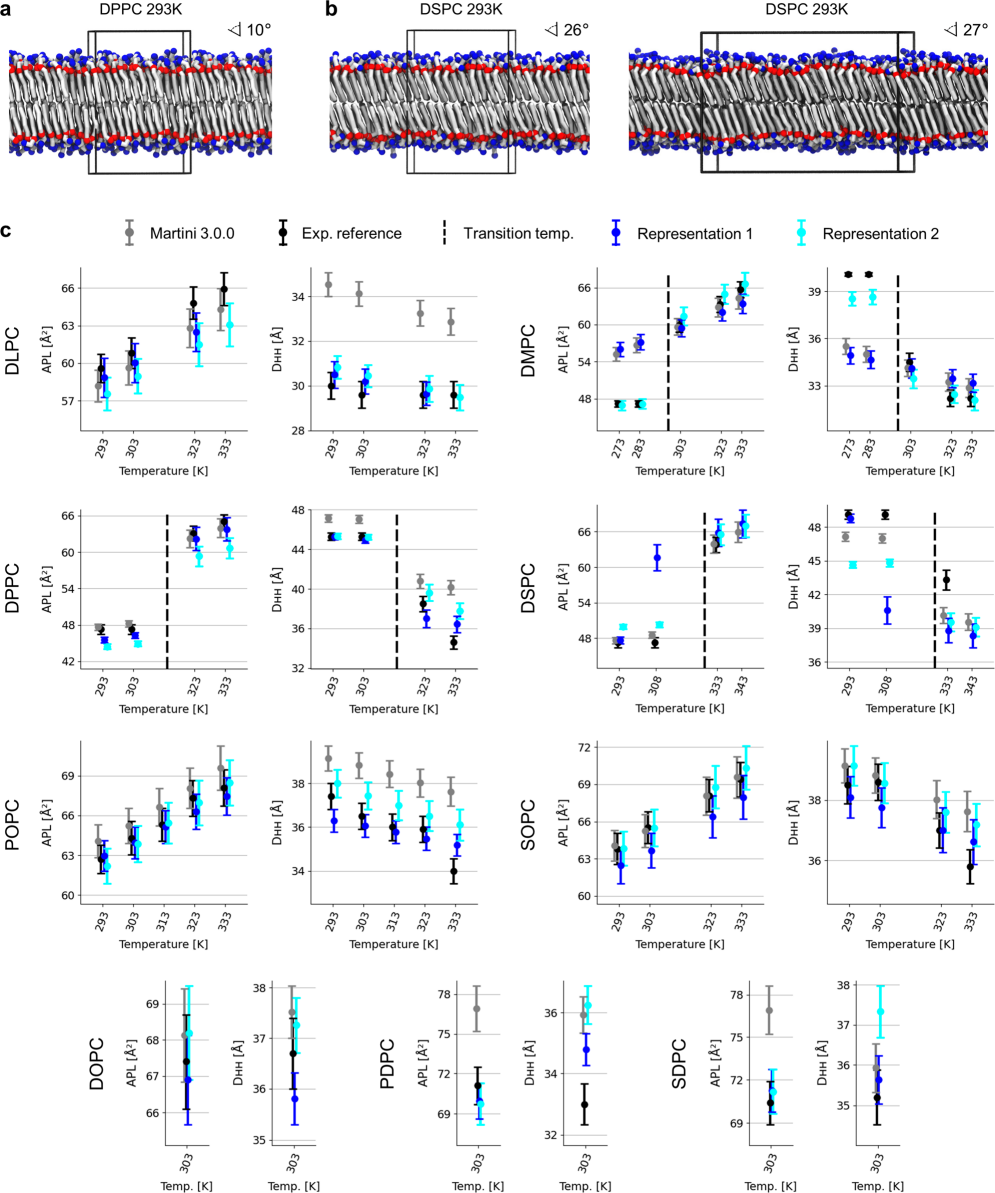
Overview of the structural properties observed for patches of lipid bilayers across multiples temperatures in the liquid and gel phases. (a) Snapshot of a DPPC bilayer simulated using Representation 2 and 128 lipids at 293 K, which exhibits moderate tails tilting. (b) Snapshots of a DSPC bilayer simulated using Representation 2 at 293 K, which exhibit significant tails tilting using either 128 lipids (left) or 512 lipids (right). (c) Summary of the APL and D_HH_ thickness values for the eight PC lipid models in the training sets and for SOPC observed experimentally (black) or in simulations using Martini 3.0.0 (gray), Representation 1 (dark blue), and Representation 2 (cyan). Dashed horizontal lines indicate the gel/liquid transition temperatures. Error bars represent ± one standard deviation (simulations) or the measurement error (experimental). Dots represent average values (simulations) and the indicated measure (experimental).

Lastly, we underline that experimental data collected for DMPC, DPPC, and DSPC in the tilted gel phase (L_β′_) correspond to measurements obtained from lamellar bilayers exhibiting non-negligible tilting of the tails with respect to the bilayer normal (∼32 degrees).^20,30^ This tilting triggers a reduction of the hydrocarbon and D_HH_ thicknesses of approximately 13–15% in the L_β′_ phase with respect to the straight gel phase (L_β_).^20,30^ Therefore, when evaluating the matching of bilayer thicknesses in between CG simulations and experimental measurements, the optimization protocol here implicitly formulates the requirement that models produce tilted tails for saturated lipids in the L_β′_ phase. In practice, not all experimental objectives can be perfectly matched when optimizing the *bonded* parameters in the context of Representation 2, and DMPC and DPPC bilayers exhibit only limited tails tilting (∼10 degrees) when simulated up to 30 K below their respective gel/liquid transition temperatures (Figure 5a). We obtain a significant tilting of the tails only for DSPC in the L_β′_ phase (Figure 5b, ∼26 degrees at 293 K; Figure 5c, reduced D_HH_ thicknesses in cyan). Although the size of the molecular systems used during optimization is small, this tilt angle is stable also with increased system size (512 lipids) in 1 μs of production simulation (Figure 5b).

In Figure 5c, we provide a summary of all APL and D_HH_ thickness measurements from extended simulations of slightly larger systems of lamellar bilayers (512 lipids each, 1 μs of production simulation) using the Martini 3.0.0^4^ lipid models compared with the parameters obtained for Representations 1 and 2, along with reference experimental data.^19,20,22,30^ Here, we focus on evaluating bilayer structural properties across phase states; hence, simulations at temperatures that correspond experimentally to gel or liquid phases are started respectively from a configuration in the tilted gel phase (L_β′_) or in the liquid phase (Section S6, Table S4). Using Representation 2, we obtain overall improved parameters with respect to Martini PC lipids version 3.0.0 in the context of this training set, which demonstrates the usefulness of such optimization protocols even when exploiting only the APL and D_HH_ thickness measurements during training. For both Representations 1 and 2, as expected, the obtained parameters also transferred well to SOPC at different temperatures in the liquid phase (Figure 5b), even if this lipid was left out of the optimization procedures. For Representation 1, however, the transition in between liquid and gel phases is not observed for DMPC and DSPC bilayers are not stable enough in the gel phase (Figure 5b). This can be associated with the usage of a mixture of small and regular beads in the lipid tails (Figure S5). For Representation 2, the transition temperatures are better respected while the behavior of lipid mixtures also appear improved as a *posterior* result of the optimization of the structural properties in pure composition bilayer simulations.

## 4 Discussion

In this study, we apply the automated optimization strategy implemented in *SwarmCG*(25,26) for the evaluation of two putative refined CG representations of the lipid models in the framework of Martini and aim at improving their structural and thermodynamic properties. Because we optimize a finite number of *bonded* parameters in an informative context (i.e., rich training sets and *nonbonded* parameters remain constant, set to Martini 3.0.0^4^), we can eliminate uncertainties related to parameters tuning for focusing on evaluating the capabilities and limits of the CG representations (i.e., the choice of beads, their reference positioning, the topologies of the CG models, and potentials used to describe *bonded* interactions). Our results indicate Representation 2 allows for overall improvement upon the current version of the PC lipid models in Martini 3.0.0^4^ (on the basis of the nine different lipid types included in the benchmark and on the objectives set), and the protocol implemented in *SwarmCG*(25,26) is effective in this context for optimizing up to 77 *bonded* parameters (and likely more). Use of mixed bead sizes in Representation 1 allows for matching of the experimental objectives well in the liquid phase and particularly for PUFA-containing lipids. The description of the phase behavior verified *a posteriori* for DOPC/DPPC mixtures appears relevant, as well, but the DPPC model in this CG representation is a special case that includes enough repeated big beads in the tails to enable the formation of a gel phase (which is not the case for DMPC and DSPC). Conclusively, the philosophy of Representation 1 cannot yield versatile building blocks for describing multiple types of lipids across phase states.

Instead, models obtained here with Representation 2 produce a more relevant modeling compromise, which allows for a better approach of all experimental objectives across phase states (Figure 5). In particular, the automated search of *bonded* parameters for the versatile building blocks of Representation 2 highlights that reproduction of the tilt angle of lipid bilayers in the L_β′_ phase in Martini simulations is possible. This is not trivial because, to our knowledge, only one example of such CG simulations exists, whereby *bonded* parameters were manually optimized initially with the goal of obtaining a relevant modeling of the tilted ripple phase (P_β′_) for DPPC,^23^ specifically, without considering the impact on the modeling of other lipid types (FF parameters are unavailable for this study). We envision that further working on the CG representation, potentially by slightly tweaking bead choices to further enhance the balance of *bonded* and *nonbonded* interactions in between lipid heads and tails, may allow for the description of tails tilting in the L_β′_ phase for relevant saturated lipids in the Martini framework.

As demonstrated, automated integrative modeling approaches, such as *SwarmCG*,^25,26^ can be successfully leveraged not only for calibrating force field parameters but also for (in)validating modeling philosophies with higher throughput and enhanced certainty than what can be done manually. To this end, we here relied principally on experimental APL and D_HH_ thickness values for optimizing the *bonded* parameters of PC lipid models by iteratively simulating small patches of bilayers at a limited number of temperatures across phase states. We note, however, that this protocol could be extended with limited efforts to obtain *bonded* building blocks for a much larger set of lipid models in the context of Martini.^4^ The training set can be further enriched by including additional bilayer systems (e.g., different lipid head types, sphingomyelins, etc.) and the loss function can also directly evaluate other experimental measurements providing transversal information (e.g., the hydrocarbon thickness would complement well the currently employed D_HH_ thickness and APL). Other available experimental properties, such as bending modulus or diffusion constants, may also be exploited at a slightly higher computational cost. The use of annealing simulations directly in the training set could also enable finer estimation and tuning of the gel/liquid phase transition temperatures.

Further developments of *SwarmCG*(25,26) may also target other classes of molecules, such as DNA, peptides, and proteins, which are well suited to the application of automatic parametrization approaches leveraging the cross-sampling of building blocks. Although the computational burden may seem important, such approaches scale well on HPC resources (N.B., in this study, we only used 64 CPU cores). Their current limits remain defined mostly by our ability to assemble rich training sets, including reliable experimental data, to be used in concert with relevant CG representations, which are sufficiently descriptive of the degrees of freedom of the molecular systems of interest.

## 5 Data and Software Availability

The code used in this study for the optimization of the CG lipid models is available at: https://github.com/GMPavanLab/SwarmCGM, together with the models obtained at convergence for each experiment, as well as the configuration files used in this study and all material necessary for reproducing the results. Complete data are also available at: 10.5281/zenodo.8010318.

## Supporting Information Available

Orthogonal view of a bilayer composed of DOPC and DPPC lipids (Representation 1) mixed at 10/90% mass at temperature 298 K in a 10 us production simulation, during which the gel and liquid phases constantly exchange lipids (MP4)
Details on the functional form of the CG FF within which the parameters are optimized; the molecular models used in these experiments, their topologies, their optimized FF parameters obtained with *SwarmCG* in the context of Representations 1 and 2, as well as the implementation for usage with HPC resources; and additional details concerning the submolecular features observed in the CG models obtained at the end of the optimization experiments, which underline the relevance of the OT-B metrics (PDF)

## ABBREVIATIONS

DLPC: 1,2-dilauroyl-*sn*-glycero-3-phosphocholine
DMPC: 1,2-dimyristoyl-*sn*-glycero-3-phosphocholine
DPPC: 1,2-dipalmitoyl-*sn*-glycero-3-phosphocholine
DSPC: 1,2-distearoyl-*sn*-glycero-3-phosphocholine
POPC: 1-palmitoyl-2-oleoyl-glycero-3-phosphocholine Δ9-Cis
SOPC: 1-stearoyl-2-oleoyl-*sn*-glycero-3-phosphocholine Δ9-Cis
DOPC: 1,2-dioleoyl-*sn*-glycero-3-phosphocholine Δ9-Cis
PDPC: 1-palmitoyl-2-docosahexaenoyl-*sn*-glycero-3-phosphocholine Δ4,7,10,13,16,19-Cis
SDPC: 1-stearoyl-2-docosahexaenoyl-*sn*-glycero-3-phosphocholine Δ4,7,10,13,16,19-Cis
DLPE: 1,2-dilauroyl-*sn*-glycero-3-phosphoethanolamine
DMPE: 1,2-dimyristoyl-*sn*-glycero-3-phosphoethanolamine
DLPS: 1,2-dilauroyl-*sn*-glycero-3-phospho-l-serine
DMPS: 1,2-dimyristoyl-*sn*-glycero-3-phospho-l-serine
