# Supplementary material for: Automatic Optimization of Lipid Models in the Martini Force Field Using SwarmCG
Source: J Chem Inf Model. 2023 Jun 6;63(12):3827–38. doi: 10.1021/acs.jcim.3c00530 (PMC10302490; doi:10.1021/acs.jcim.3c00530)
Supplement: Supplementary file 2 — ci3c00530_si_002.pdf [file ci3c00530_si_002.pdf]

Supplementary material for:

Automatic Optimization of Lipid Models in the Martini Force Field Using SwarmCG

Charly Empereur-mot,[a,*] Kasper B. Pedersen,[b] Riccardo Capelli,[c] Martina Crippa,[d] Cristina Caruso,[d] Mattia Perrone,[d] Paulo C.T. Souza,[e] Siewert J. Marrink[f] & Giovanni M. Pavan[a,d,*]

[a] *Department of Innovative Technologies, University of Applied Sciences and Arts of Southern Switzerland, Polo Universitario Lugano, Campus Est, Via la Santa 1, 6962 Lugano-Viganello, Switzerland*

[b] *Department of Chemistry, Aarhus University, Langelandsgade 140, 8000 Aarhus C, Denmark*

[c] *Department of Biosciences, Università degli Studi di Milano, Via Celoria 26, 20133 Milano, Italy*

[d] *Politecnico di Torino, Department of Applied Science and Technology, Corso Duca degli Abruzzi 24, 10129 Torino, Italy*

[e] *Molecular Microbiology and Structural Biochemistry (MMSB, UMR 5086), CNRS & University of Lyon, 7 Passage du Vercors, 69007 Lyon, France*

[f] *Molecular Dynamics, Groningen Biomolecular Sciences and Biotechnology Institute (GBB), University of Groningen, Nijenborgh 7, 9747 AG Groningen, The Netherlands*

[*] *Corresponding authors:* charly.empereur-mot@supsi.ch, giovanni.pavan@polito.it

S1. Functional form of the coarse-grain force field

The functional form of the coarse-grain (CG) force field (FF) employed in this study include *bonded* and *non-bonded* interaction potentials. We optimize exclusively the *bonded* interaction terms, while the *non-bonded* interaction terms remain constant (set to Martini 3.0.0[1]). The *bonded* interactions are described using harmonic potentials for bonds and angles (no dihedral potentials are applied) in the forms

$$U_{bond}(d) = \frac{1}{2}k_{bond}(d - d_0)^2, \tag{S1}$$

where d is the distance between 2 covalently bound particles, k_{bond} is the force constant applied for this type of bond and d_0 is its equilibrium value; and

$$U_{angle}(\theta) = \frac{1}{2}k_{angle}(\theta - \theta_0)^2, \tag{S2}$$

where θ is the angle between 3 successive particles, k_{angle} is the force constant applied for this type of angle and θ_0 is its equilibrium value. The *non-bonded* interactions are described using Lennard-Jones (LJ) and Coulomb potentials in the forms

$$V_{LJ}(r) = 4\varepsilon_{i,j}\left[\left(\frac{\sigma_{i,j}}{r}\right)^{12} - \left(\frac{\sigma_{i,j}}{r}\right)^{6}\right], \tag{S3}$$

where $\sigma_{i,j}$ represents the closest distance allowed between 2 particles (beads) of types i and j, $\varepsilon_{i,j}$ is their interaction strength and r is the distance between these 2 particles; and

$$V_{Coulomb}(r) = \frac{q_i q_j}{4\pi\varepsilon_0\varepsilon_r r}, \tag{S4}$$

where q_i and q_j represent the charge of each particle, ε_0 is the electric constant and ε_r is the relative dielectric constant used for explicit screening, that is here set to 15 in the framework of Martini[1]. The functional form of the CG FFs is then

$$W = U_{bonds} + U_{angles} + V_{LJs} + V_{Coulombs}. \tag{S5}$$

S2. Reference measurements from experiments and available force fields (APL & DHH)

In this section, we provide APL and DHH measurements obtained using different reference AA and CG FFs via MD simulations of small patches of lipid bilayers (128 lipids, 64 per leaflet) and compare these values to reference experimental measurements obtained via small-angle neutron scattering (SANS) and small-angle X-ray scattering (SAXS) using lamellar bilayer isolates[2–5] (Table S1). We perform this benchmarking step in order to select the AA FF that will provide the best accuracy for the experimental data we selected as target (area per lipid & DHH thickness) and for the couples of lipid types and temperatures to be employed in the optimization procedures. Our conclusion from the AA MD simulations which measurements are shown in Table S1 for phosphatidylcholine (PC) lipids, using state-of-the-art AA FFs, is that there currently

exists no AA FFs accurately describing the global structural properties of lipid bilayers for systems composed of lipids including polyunsaturated fatty acids (*i.e.* for SDPC and PDPC, large errors on APL and/or D_{HH}). For Charmm36 LJ-PME[6,7], we simulated only homogeneous bilayers composed of DLPC, POPC or SDPC, as this is sufficient to characterize the improvement as marginal, if any, compared to Charmm36[8] in terms of average APL and D_{HH} for the lipids and temperatures used in this study. Charmm36[8] has already been described as accurate for the simulation of both gel and liquid phases of saturated PC lipids[9], which we could confirm (Table S1). We selected Charmm36[8] for running the AA simulations used as the *bottom-up* reference in this study.

S2.1. All-atom force fields

All AA MD simulations used for measuring APL and D_{HH} in Table S1 were conducted using GROMACS 2020.4[10,11]. AA bilayer systems were created using Charmm-GUI[12], positioning the plane of the bilayers perpendicularly to the Z-axis of the MD simulation box. All the systems were first energy-minimized using the steepest descent algorithm (5000 steps), followed by an equilibration run of 500 ns using an integration step of 2 fs. The temperature was maintained at the values indicated in Table S1 and the pressure was set to P = 1 bar by coupling the dynamics, respectively, with the V-rescale thermostat[13] (τ_t = 1 ps^{-1}) and the Parrinello-Rahman barostat[14] (τ_p = 20 ps^{-1}, compressibility = 4.5e^{-5} bar^{-1}), with semiisotropic pressure scaling. In production runs, the dynamics was integrated for 1 μs (1000 frames) using instead the Parrinello-Rahman barostat[14] (compressibility = 4.5e^{-5} bar^{-1}, τ_p = 5 ps^{-1} for Slipids[15], τ_p = 20 ps^{-1} for Charmm36[8] and Charmm36 LJ-PME[6,7]), while other MD parameters are unchanged with respect to the equilibration runs. In the case of Charmm36 LJ-PME[6,7], long range electrostatics is handled using the Particle Mesh Ewald (PME) method[16]. The cut-offs used for Van der Waals and Coulomb interactions are set to 1.5, 1.2 and 1.0 nm for Slipids[15], Charmm36[8] and Charmm36 LJ-PME[6,7], respectively. The APL measurements are obtained by dividing the size of the X-side of the MD simulation box by half the number of lipids constituting the bilayer ($128/2$). The D_{HH} thickness measurements were obtained using the position of the phosphate atom in the lipids heads. APL and D_{HH} values shown in Table S1 are average measurements from the production runs.

Lipid	Temp.	Experimental APL [Å²]	Experimental DHH [Å]	Slipids[15] APL (std) [Å²]	ΔAPL [%]	DHH (std) [Å]	ΔDHH [%]
DLPC	303K	60.8	29.8	63.2 (1.2)	+ 3.9	30.1 (0.5)	+ 0.9
DMPC	273K	47.2	40.1	-	-	-	-
DMPC	303K	59.8	34.9	61.5 (1.2)	+ 2.9	34.3 (0.6)	- 1.7
DMPC	323K	63.3	32.2	-	-	-	-
DPPC	293K	47.3	45.3	-	-	-	-
DPPC	323K	63.1	38.6	63.5 (1.4)	+ 0.8	37.3 (0.70)	- 3.3
DSPC	308K	47.3	49.1	-	-	-	-
DSPC	333K	63.8	43.3	-	-	-	-
POPC	303K	64.3	36.5	65.9 (1.2)	+ 2.5	37.1 (0.6)	+ 1.5
DOPC	303K	67.4	36.8	69.1 (1.2)	+ 2.6	36.9 (0.6)	+ 0.2
SDPC	303K	70.4	35.2	64.5 (1.7)	- 8.4	39.6 (1.2)	+ 12.5
PDPC	303K	71.1	33.0	65.8 (1.5)	- 7.4	37.4 (0.8)	+ 13.2

Table S1. APL and DHH measurements obtained from bilayer isolates experimentally and from MD simulations (average measures) using the Slipids[15], Charmm36[8] and Charmm36 LJ-PME[6,7] AA FFs. Standard deviations are shown in parenthesis. Some simulations were performed exclusively for benchmarking purposes and exclusively Charmm36[8] was used as reference in this study.

Lipid	Temp.	Charmm36[8] APL (std) [Å²]	ΔAPL [%]	DHH (std) [Å]	ΔDHH [%]	Charmm36 LJ-PME[6,7] APL (std) [Å²]	ΔAPL [%]	DHH (std) [Å]	ΔDHH [%]
DLPC	303K	62.5 (1.2)	+ 2.7	31.4 (0.5)	+ 5.5	63.83 (1.44)	+ 4.99	30.68 (0.52)	+ 2.94
DMPC	273K	50.2 (0.5)	+ 6.4	38.8 (0.6)	- 3.2	-	-	-	-
DMPC	303K	59.8 (1.2)	- 0.1	36.1 (0.6)	+ 3.3	-	-	-	-
DMPC	323K	64.3 (1.3)	+ 1.6	33.5 (0.6)	+ 4.1	-	-	-	-
DPPC	293K	51.4 (1.0)	+ 8.7	42.8 (0.6)	- 5.5	-	-	-	-
DPPC	323K	60.8 (1.4)	- 3.6	39.7 (0.7)	+ 2.8	-	-	-	-
DSPC	308K	49.6 (0.6)	- 4.7	46.8 (0.5)	- 4.6	-	-	-	-
DSPC	333K	60.8 (1.3)	- 4.8	43.6 (0.7)	+ 0.8	-	-	-	-
POPC	303K	64.9 (1.3)	+ 0.9	38.6 (0.6)	+ 5.9	64.9 (1.2)	+ 0.9	38.3 (0.5)	+ 5.0
DOPC	303K	68.2 (1.3)	+ 1.1	38.5 (0.6)	+ 4.6	-	-	-	-
SDPC	303K	69.4 (1.4)	- 1.4	39.8 (0.7)	+ 12.9	70.0 (1.4)	- 0.5	39.2 (0.7)	+ 11.3
PDPC	303K	69.6 (1.5)	- 2.1	38.1 (0.7)	+ 15.5	-	-	-	-

Table S1 (continued). APL and DHH measurements obtained from bilayer isolates experimentally and from MD simulations (average measures) using the Slipids[15], Charmm36[8] and Charmm36 LJ-PME[6,7] AA FFs. Standard deviations are shown in parenthesis. Some simulations were performed exclusively for benchmarking purposes and exclusively Charmm36[8] was used as reference in this study.

S2.2. Coarse-grained force fields

All CG MD simulations used for measuring APL and DHH in Table S2 were performed using GROMACS 2020.4[10,11]. CG bilayer systems were created using Charmm-GUI[12], positioning the plane of the bilayers orthogonal to the Z-axis of the MD simulation box. All the systems were first energy-minimized using the steepest descent algorithm (5000 steps), followed by an equilibration run of 500 ns using an integration step of 20 fs. The temperature was maintained at the values indicated in Table S2 and the pressure was set to P = 1 bar by coupling the dynamics, respectively, with the V-rescale thermostat[13] (τ_t = 1 ps^{-1}) and the Parrinello-Rahman barostat[14] (τ_p = 12 ps^{-1}, compressibility = 3e^{-4} bar^{-1}), with semiisotropic pressure scaling. In production runs, the dynamics was integrated for 1 µs (1000 frames) using instead an integration step of 20 fs and the Parrinello-Rahman barostat[14] (compressibility = 3e^{-4} bar^{-1}, τ_p = 12 ps^{-1}), while other MD parameters are unchanged with respect to the equilibration runs. The cut-offs used for Van der Waals and Coulomb interactions are set to 1.1 nm and the relative dielectric constant is set to 15, according to the Martini[1] framework. The APL measurements are obtained by dividing the size of the X-side of the MD simulation box by half the number of lipids constituting the bilayer. The DHH thickness measurements were obtained using the position of the bead that includes the (mapped) phosphate atom in the lipid heads. Additionally, the DHH thickness delta between the AA phosphate position and the effective position of the CG bead containing the phosphate was calculated from AA and AA-mapped reference trajectories, respectively, and accounted for in the CG DHH calculations. APL and DHH values shown in Table S2 are average measurements from the production runs.

		Experimental		Martini 3.0.0[1]			
**Lipid**	**Temp.**	**APL [Å²]**	**DHH [Å]**	**APL (std) [Å²]**	**ΔAPL [%]**	**DHH (std) [Å]**	**ΔDHH [%]**
**DLPC**	**303K**	60.8	29.8	59.7 (1.3)	- 1.9	34.1 (0.6)	+ 14.5
**DMPC**	**273K**	47.2	40.1	55.2 (1.1)	+ 16.9	35.5 (0.5)	- 11.5
**DMPC**	**303K**	59.8	34.9	59.7 (1.4)	- 0.2	34.1 (0.5)	- 2.3
**DMPC**	**323K**	63.3	32.2	62.8 (1.5)	- 0.8	33.3 (0.6)	+ 3.4
**DPPC**	**293K**	47.3	45.3	56.4 (1.1)	+ 19.2	43.2 (0.6)	- 4.6
**DPPC**	**323K**	63.1	38.6	62.2 (1.5)	- 1.4	40.8 (0.7)	+ 5.7
**DSPC**	**308K**	47.3	49.1	61.7 (2.2)	+ 30.4	40.6 (1.2)	- 17.3
**DSPC**	**333K**	63.8	43.3	65.8 (2.3)	+ 3.1	38.8 (1.1)	- 10.4
**POPC**	**303K**	64.3	36.5	65.3 (1.3)	+ 1.6	38.8 (0.6)	+ 6.3
**DOPC**	**303K**	67.4	36.8	68.1 (1.3)	+ 1.0	37.5 (0.6)	+ 1.9
**SDPC**	**303K**	70.4	35.2	76.9 (1.7)	+ 9.2	35.9 (0.6)	+ 2.0
**PDPC**	**303K**	71.1	33.0	76.9 (1.7)	+ 8.2	35.9 (0.6)	+ 8.8

Table S2. APL and DHH measurements obtained from lamellar bilayers experimentally and from MD simulations (average measures) using the Martini 3.0.0 CG FF. Standard deviations are shown in parenthesis.

S3. Measurements obtained from iterative CG simulations during optimization (APL, D_{HH} & OT-B)

All CG MD simulations were again performed using GROMACS 2020.4[10,11]. CG bilayer systems for lipid molecules in Representation 1 and 2 were created by first mapping a frame from reference AA trajectories, at the relevant temperatures used in the optimization procedures. For lipid types which optimization targets include both liquid and gel phase states (DMPC, DPPC and DSPC) a frame was selected instead from an AA simulation in the ripple phase ($P_{\beta'}$), in order to limit bias in the later evolution of the systems towards either the liquid or gel phase. Simulations in the ripple phase were performed using parameters identical to those cited in Section S2.1 at temperatures of 293K for DMPC, 311K for DPPC and 325K for DSPC[9]. After mapping the lipids in these frames to CG resolution, these were centered in their simulation boxes after modifying their size on the Z-axis (orthogonal to the bilayer) to 13 nm. Systems were solvated using GROMACS command 'solvate' with argument 'solv_radius' set to 0.23 nm and providing a box of Martini 3.0.0 water (bead type 'W') equilibrated at the corresponding temperature. Lastly, a small amount of ions was added to the systems (1 ion per 8 lipid molecules) to avoid water freezing at low temperatures. The composition of all the CG systems simulated iteratively in this study is provided in Table S3. The CG simulation parameters are identical to those cited in section S2.2. except for the equilibration times that are set to 200 ns and 50 ns, respectively, for simulation in the gel or liquid phases. The production times, used for measuring APL, D_{HH} thickness and OT-B metrics are always set to 200 ns. The domains of the bond and angle distributions used for OT-B calculations[17] are set to [0, 50] Å and [0, 180] degrees, respectively, for the distributions of all AA-mapped and CG bond and angle, with histogram bandwidths set to 0.1 Å and 2 degrees. The domain of all the AA-mapped and CG distance distributions between pairs of bead types is set to [0, 15] Å and the bandwidth used is 0.25 Å.

	Representation 1				Representation 2			
**Lipid**	**Lipids**	**Water**	**Na**	**Cl**	**Lipids**	**Water**	**Na**	**Cl**
**DLPC**	128	2846	8	8	128	2846	8	8
**DMPC**	128	2182	8	8	128	2180	8	8
**DPPC**	128	1917	8	8	128	1915	8	8
**DSPC**	128	1667	8	8	128	1765	8	8
**POPC**	128	2711	8	8	128	2708	8	8
**DOPC**	128	2915	8	8	128	2914	8	8
**SDPC**	128	2634	8	8	128	2633	8	8
**PDPC**	128	2706	8	8	128	2702	8	8

Table S3. Composition of the systems simulated iteratively during the optimization of the *bonded* parameters used in Representations 1 and 2 (number of lipids, water beads, sodium ion beads and chloride ion beads). Systems used across different temperatures have identical composition.

S4. Details on the topologies of Representation 1

For the optimization performed using Representation 1 (Fig. 2 of main text), we optimize the following 77 parameters corresponding to building blocks of the *bonded* CG FF shown in Figure S1:

- equilibrium values and force constants for bond types B1 to B16 (32 parameters),
- equilibrium values for angle types A2 to A6, A11 and A15 to A26 (18 parameters),
- force constants for angle types A1 to A27 (27 parameters).

The equilibrium value and force constant of a bond correspond respectively to parameters d and k_{bond} in Equation S1. The equilibrium value and force constant of an angle correspond respectively to parameters θ and k_{angle} in Equation S2. Non-bonded interaction parameters from the Martini 3.0.0[1] FF were applied and are not optimized here. Equilibrium value remains at 180° for angle types A1, A7 to 10, A12 to 14 and A27, which correspond to angles for which we observed distribution average close to 170-180° in reference AA-mapped trajectories.

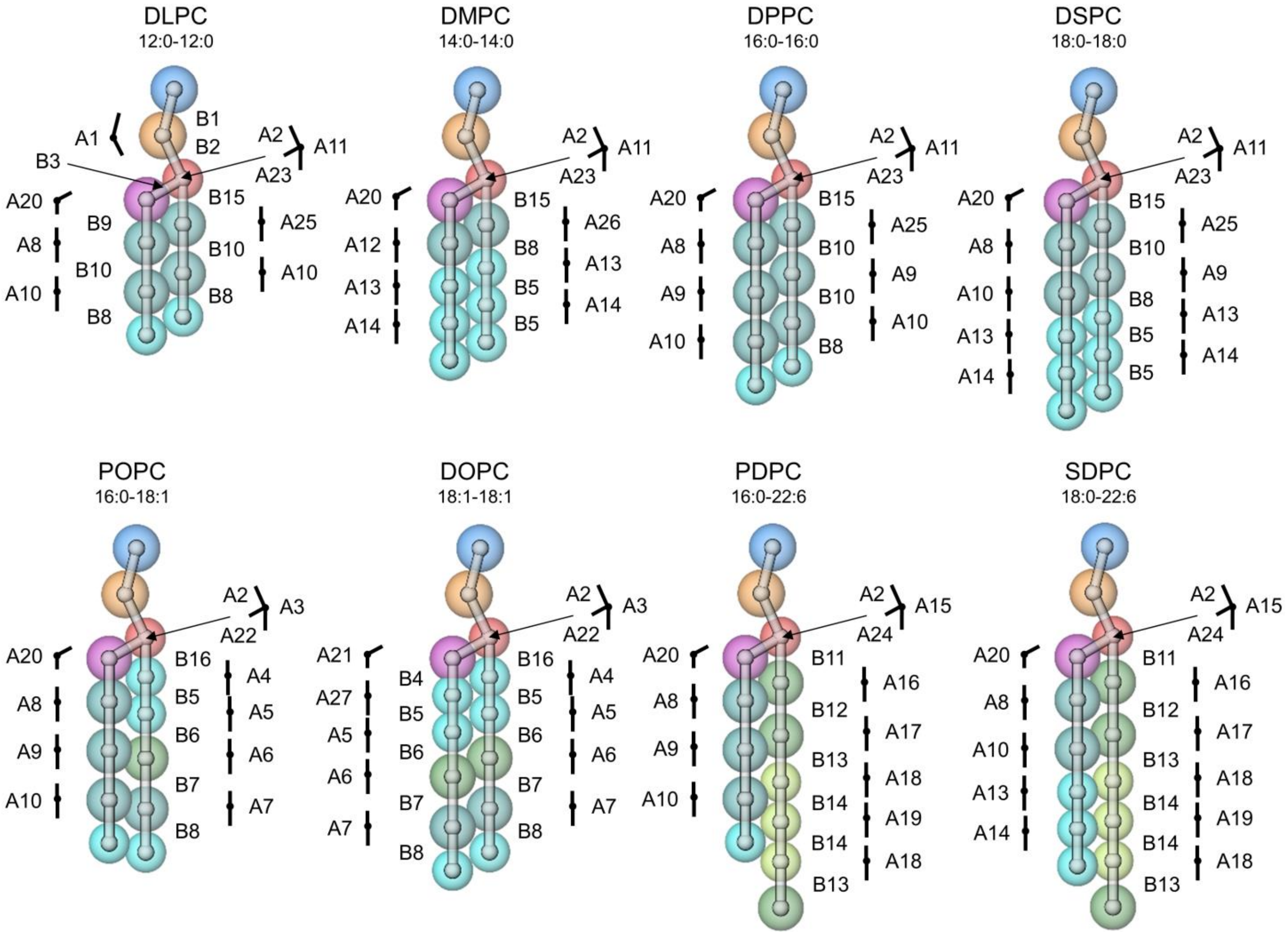

Figure S1. Overview of the CG representations and typing of the *bonded* topology elements used for Representation 1 in this study (Bond types: B*n*, Angle types: A*n*). Bead types applied: Q1 (dark blue), Q5 (orange), SN4a (red), N4a (purple), C1 (blue), SC2 (cyan), C4h (olive) and SC4h (bright yellow/green). Too redundant bond and angle types in the head group or tails are sometimes hidden for clarity.

To support the fact that the bottom-up component of the loss function effectively allows to guide the optimization towards reproducing within CG models the structural features present in AA-mapped trajectories, we show here the bond and angle distributions obtained in bilayers from the optimized DLPC, POPC and SDPC models simulated at 303K in Figure 2 of main text. The CG *vs.* AA-mapped angle distributions are well aligned overall (Fig. S2-4).

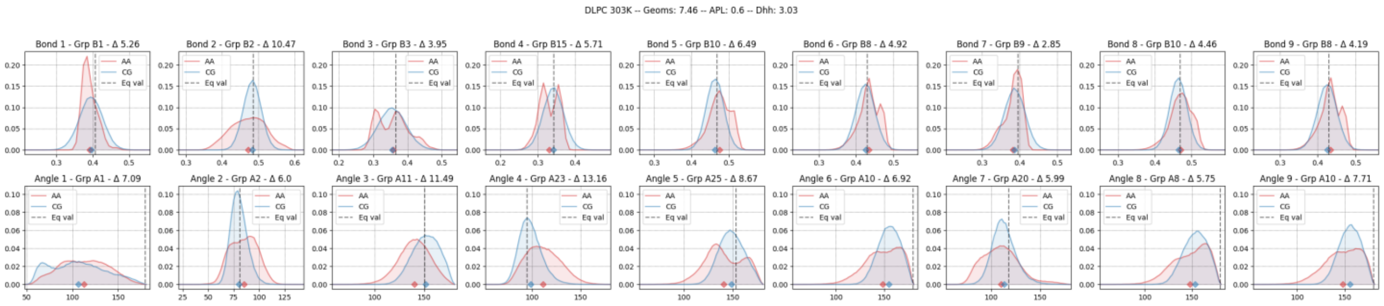

Figure S2. Bond and angle distributions obtained in bilayer simulation at 303K for the CG model of DLPC with Representation 1 with super-imposed distributions from the reference AA-mapped trajectory for this lipid.

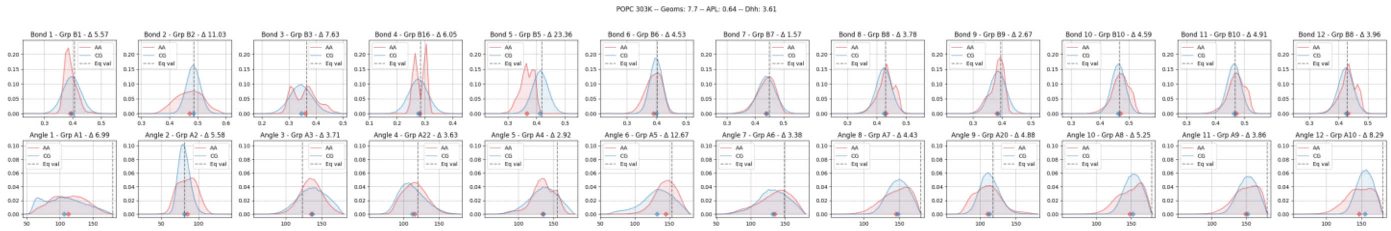

Figure S3. Bond and angle distributions obtained in bilayer simulation at 303K for the CG model of POPC with Representation 1 with super-imposed distributions from the reference AA-mapped trajectory for this lipid.

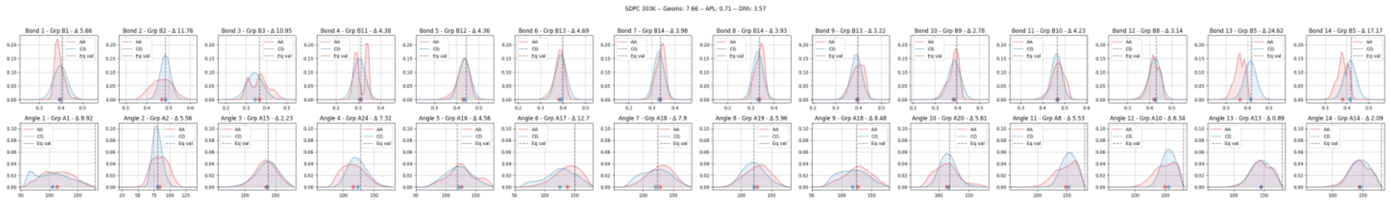

Figure S4. Bond and angle distributions obtained in bilayer simulation at 303K for the CG model of SDPC with Representation 1 with super-imposed distributions from the reference AA-mapped trajectory for this lipid.

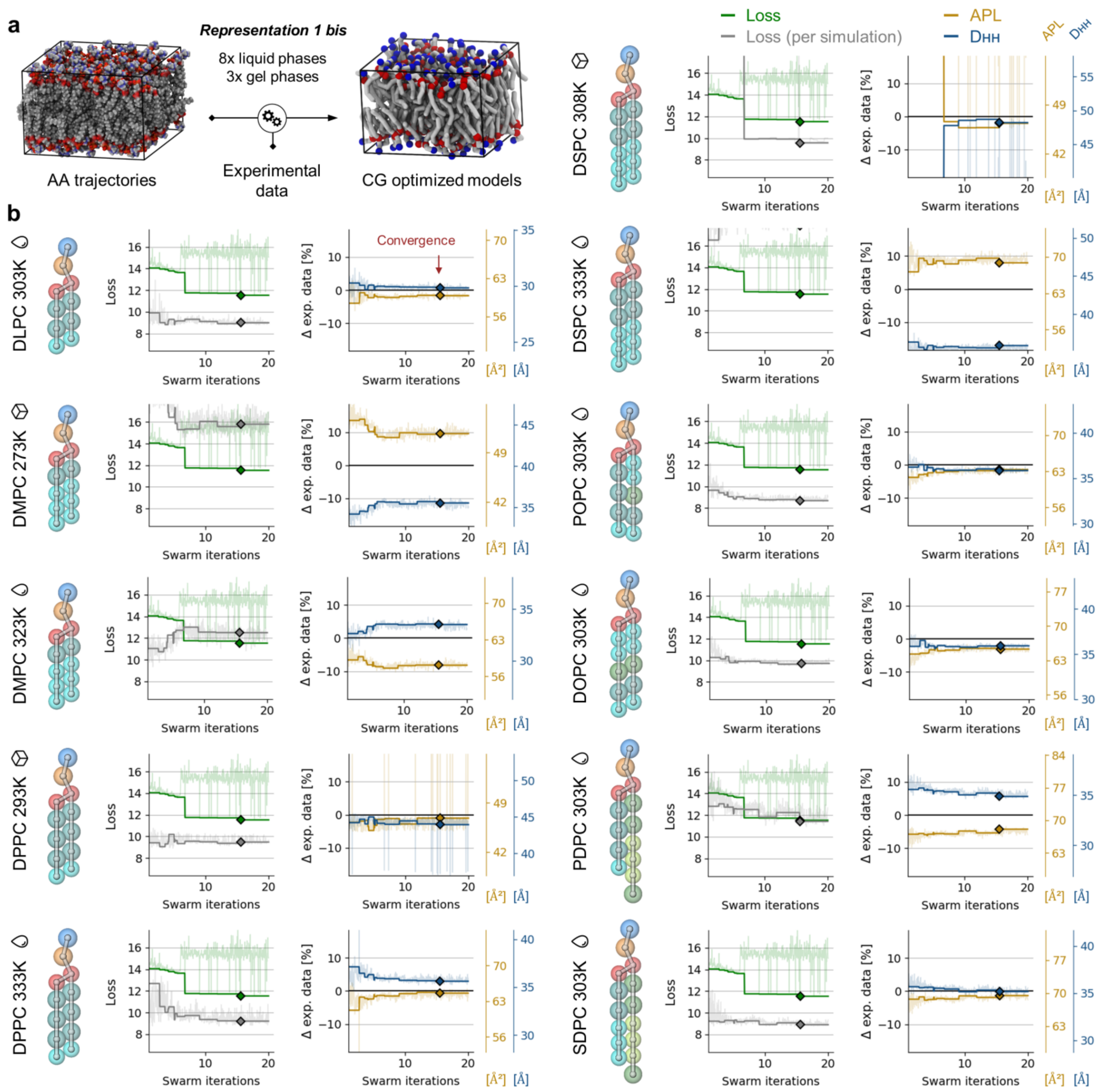

Figure S5. Multi-objective optimization of the *bonded* parameters of the FF for PC lipid models built in the framework of Martini 3.0.0 using Representation 1 and in the training set bilayers of 8 different lipid types simulated at 11 temperatures (DLPC 303K, DMPC 273K & 323K, DPPC 293K & 333K, DSPC 308K & 333K, POPC 303K, DOPC 303K, PDPC 303K & SDPC 303K). (a) Illustration summarizing the workflow. (b) Left panels: loss global (green) and loss per bilayer simulation (grey) in the training set. Right panels: APL (yellow) and DHH (blue) for each bilayer simulation in the training set. The horizontal black lines set at 0 identify the target experimental APL and DHH values. Solid curves are values corresponding to the best global loss at any point during optimization. Shaded lines show raw data. Diamonds represent values at convergence, obtained with the optimized *bonded* parameters. The drop and box icons respectively represent the liquid and gel states of pure lipid bilayers at the corresponding temperatures.

S5. Details on the topologies of Representation 2

For the optimization performed using Representation 2 (Fig. 4 of main text), we optimize the following 48 parameters corresponding to building blocks of the *bonded* CG FF:

- equilibrium values and force constants for bond types B1 to B13 (26 parameters),
- equilibrium values for angle types A2 to A9, A11 and A12 (10 parameters),
- force constants for angle types A1 to A12 (12 parameters).

The equilibrium value and force constant of a bond correspond respectively to parameters d and k_{bond} in Equation S1. The equilibrium value and force constant of an angle correspond respectively to parameters θ and k_{angle} in Equation S2. Non-bonded interaction parameters from the Martini 3.0.0[1] FF were applied and are not optimized here. Equilibrium value remains at 180° for angle types A1 and A10, which correspond to angles in saturated tails.

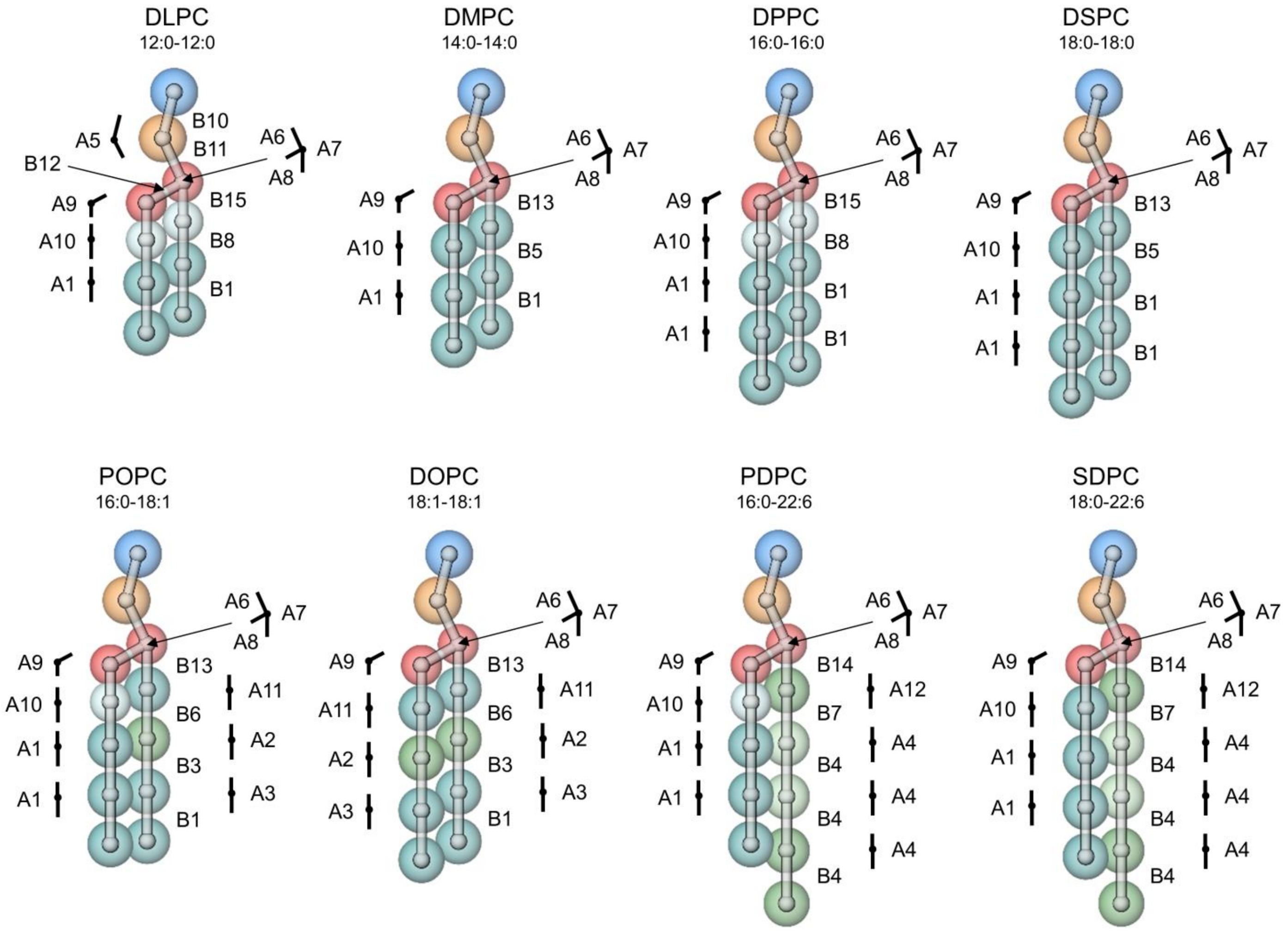

Figure S6. Overview of the CG representations and typing of the *bonded* topology elements used for Representation 1 in this study (Bond types: B*n*, Angle types: A*n*). Bead types applied: Q1 (dark blue), Q5 (orange), SN4a (red), C1 (blue), SC1 (white), C4h (olive) and C5h (light green). Too redundant bond and angle types in the head group or tails are sometimes hidden for clarity.

To support the fact that the bottom-up component of the loss function effectively allows to guide the optimization towards reproducing within CG models the structural features present in AA-mapped trajectories, we show here the bond and angle distributions obtained in bilayers from the optimized DLPC, POPC and SDPC models simulated at 303K in Figure 4 of main text. The CG *vs.* AA-mapped angle distributions are well aligned overall (Fig. S7-9).

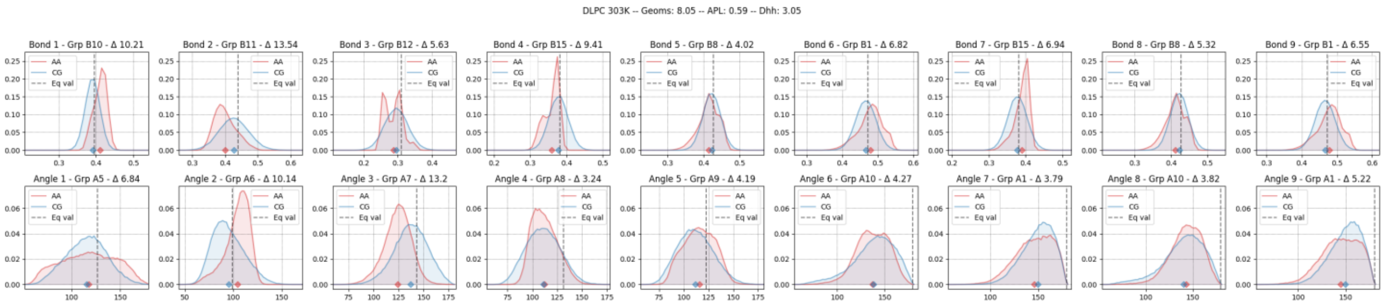

Figure S7. Bond and angle distributions obtained in bilayer simulation at 303K for the CG model of DLPC with Representation 2 with super-imposed distributions from the reference AA-mapped trajectory for this lipid.

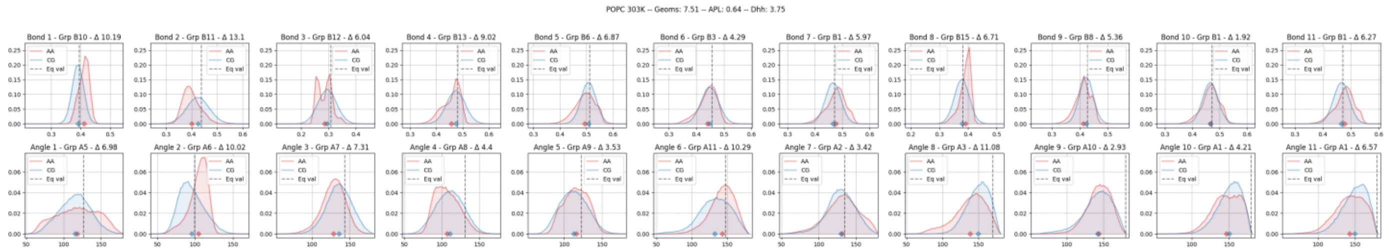

Figure S8. Bond and angle distributions obtained in bilayer simulation at 303K for the CG model of POPC with Representation 2 with super-imposed distributions from the reference AA-mapped trajectory for this lipid.

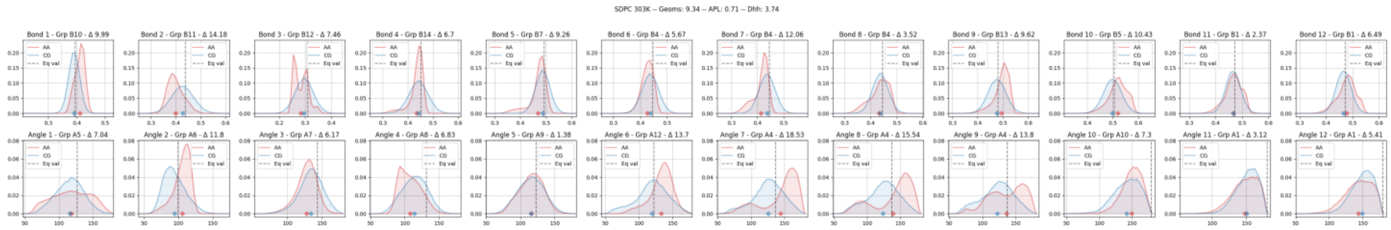

Figure S9. Bond and angle distributions obtained in bilayer simulation at 303K for the CG model of SDPC with Representation 2 with super-imposed distributions from the reference AA-mapped trajectory for this lipid.

S6. Measurements obtained from *posterior* CG simulations (APL & D_{HH})

All CG MD simulations were again performed using GROMACS 2020.4[10,11]. CG bilayer systems were created by replicating the systems from Sections S2.2 and S3 to create systems of 512 lipids from the initial ones with 128 lipids. The CG simulation parameters are identical as those cited in section S2.2. except for the equilibration and production times that are set to 500 ns and 1 μs. Long simulations are sometimes required to observe gel/liquid phase transitions in bilayer systems, notably depending on system size[1]. Where necessary for obtaining the relevant starting configurations of the bilayers in the gel or liquid phase, we triggered the formation of a gel phase by lowering the temperature by 20-30K in an intermediate simulation. The composition of the systems used for *posterior* validation for Representations 1 and 2 corresponds to those in Table S3 to which a factor 4 needs to be applied. The composition of the SOPC systems for Representation 1 and 2 is identical to the one of DOPC in each representation (used as a basis for creation). The composition of the Martini 3.0.0 systems used for *posterior* evaluation is provided in Table S4.

	Martini 3.0.0[1]			
**Lipid**	**Lipids**	**Water**	**Na**	**Cl**
**DLPC/DMPC**	512	4068	8	8
**DPPC/DSPC**	512	7668	8	8
**POPC/SOPC**	512	8804	8	8
**DOPC**	512	8580	8	8
**SDPC/PDPC**	512	10036	8	8

Table S4. Composition of the systems simulated as part of the *posterior* evaluation and comparison with Martini 3.0.0 (number of lipids, water beads, sodium ion beads and chloride ion beads). Systems used across different temperatures have identical composition.

SUPPLEMENTARY REFERENCES

(1) Souza, P. C. T.; Alessandri, R.; Barnoud, J.; Thallmair, S.; Faustino, I.; Grünewald, F.; Patmanidis, I.; Abdizadeh, H.; Bruininks, B. M. H.; Wassenaar, T. A.; Kroon, P. C.; Melcr, J.; Nieto, V.; Corradi, V.; Khan, H. M.; Domański, J.; Javanainen, M.; Martinez-Seara, H.; Reuter, N.; Best, R. B.; Vattulainen, I.; Monticelli, L.; Periole, X.; Tieleman, D. P.; de Vries, A. H.; Marrink, S. J. Martini 3: A General Purpose Force Field for Coarse-Grained Molecular Dynamics. *Nat. Methods* **2021**, *18* (4), 382–388. https://doi.org/10.1038/s41592-021-01098-3.

(2) Marquardt, D.; Heberle, F. A.; Pan, J.; Cheng, X.; Pabst, G.; Harroun, T. A.; Kučerka, N.; Katsaras, J. The Structures of Polyunsaturated Lipid Bilayers by Joint Refinement of Neutron and X-Ray Scattering Data. *Chem. Phys. Lipids* **2020**, *229*, 104892. https://doi.org/10.1016/j.chemphyslip.2020.104892.

(3) Kučerka, N.; Nieh, M.-P.; Katsaras, J. Fluid Phase Lipid Areas and Bilayer Thicknesses of Commonly Used Phosphatidylcholines as a Function of Temperature. *Biochim. Biophys. Acta BBA - Biomembr.* **2011**, *1808* (11), 2761–2771. https://doi.org/10.1016/j.bbamem.2011.07.022.

(4) Tristram-Nagle, S.; Liu, Y.; Legleiter, J.; Nagle, J. F. Structure of Gel Phase DMPC Determined by X-Ray Diffraction. *Biophys. J.* **2002**, *83* (6), 3324–3335. https://doi.org/10.1016/S0006-3495(02)75333-2.

(5) Nagle, J. F.; Cognet, P.; Dupuy, F. G.; Tristram-Nagle, S. Structure of Gel Phase DPPC Determined by X-Ray Diffraction. *Chem. Phys. Lipids* **2019**, *218*, 168–177. https://doi.org/10.1016/j.chemphyslip.2018.12.011.

(6) Yu, Y.; Krämer, A.; Venable, R. M.; Brooks, B. R.; Klauda, J. B.; Pastor, R. W. CHARMM36 Lipid Force Field with Explicit Treatment of Long-Range Dispersion: Parametrization and Validation for Phosphatidylethanolamine, Phosphatidylglycerol, and Ether Lipids. *J. Chem. Theory Comput.* **2021**, *17* (3), 1581–1595. https://doi.org/10.1021/acs.jctc.0c01327.

(7) Yu, Y.; Krämer, A.; Venable, R. M.; Simmonett, A. C.; MacKerell, A. D.; Klauda, J. B.; Pastor, R. W.; Brooks, B. R. Semi-Automated Optimization of the CHARMM36 Lipid Force Field to Include Explicit Treatment of Long-Range Dispersion. *J. Chem. Theory Comput.* **2021**, *17* (3), 1562–1580. https://doi.org/10.1021/acs.jctc.0c01326.

(8) Best, R. B.; Zhu, X.; Shim, J.; Lopes, P. E. M.; Mittal, J.; Feig, M.; MacKerell, A. D. Optimization of the Additive CHARMM All-Atom Protein Force Field Targeting Improved Sampling of the Backbone φ, ψ and Side-Chain X1 and X2 Dihedral Angles. *J. Chem. Theory Comput.* **2012**, *8* (9), 3257–3273. https://doi.org/10.1021/ct300400x.

(9) Khakbaz, P.; Klauda, J. B. Investigation of Phase Transitions of Saturated Phosphocholine Lipid Bilayers via Molecular Dynamics Simulations. *Biochim. Biophys. Acta BBA - Biomembr.* **2018**, *1860* (8), 1489–1501. https://doi.org/10.1016/j.bbamem.2018.04.014.

(10) Berendsen, H. J. C.; van der Spoel, D.; van Drunen, R. GROMACS: A Message-Passing Parallel Molecular Dynamics Implementation. *Comput. Phys. Commun.* **1995**, *91* (1), 43–56. https://doi.org/10.1016/0010-4655(95)00042-E.

(11) Abraham, M. J.; Murtola, T.; Schulz, R.; Páll, S.; Smith, J. C.; Hess, B.; Lindahl, E. GROMACS: High Performance Molecular Simulations through Multi-Level Parallelism from Laptops to Supercomputers. *SoftwareX* **2015**, *1–2*, 19–25. https://doi.org/10.1016/j.softx.2015.06.001.

(12) Jo, S.; Kim, T.; Iyer, V. G.; Im, W. CHARMM-GUI: A Web-Based Graphical User Interface for CHARMM. *J. Comput. Chem.* **2008**, *29* (11), 1859–1865. https://doi.org/10.1002/jcc.20945.

(13) Bussi, G.; Donadio, D.; Parrinello, M. Canonical Sampling through Velocity Rescaling. *J. Chem. Phys.* **2007**, *126* (1), 014101. https://doi.org/10.1063/1.2408420.

(14) Parrinello, M.; Rahman, A. Polymorphic Transitions in Single Crystals: A New Molecular Dynamics Method. *J. Appl. Phys.* **1981**, *52* (12), 7182. https://doi.org/10.1063/1.328693.

(15) Grote, F.; Lyubartsev, A. P. Optimization of Slipids Force Field Parameters Describing Headgroups of Phospholipids. *J. Phys. Chem. B* **2020**, *124* (40), 8784–8793. https://doi.org/10.1021/acs.jpcb.0c06386.

(16) Darden, T.; York, D.; Pedersen, L. Particle Mesh Ewald: An N·log(N) Method for Ewald Sums in Large Systems. *J. Chem. Phys.* **1993**, *98* (12), 10089–10092. https://doi.org/10.1063/1.464397.

(17) Empereur-mot, C.; Capelli, R.; Perrone, M.; Caruso, C.; Doni, G.; Pavan, G. M. Automatic Multi-Objective Optimization of Coarse-Grained Lipid Force Fields Using SwarmCG. *J. Chem. Phys.* **2022**, *156* (2), 024801. https://doi.org/10.1063/5.0079044.
